# Photochemistry of Flavonoids

**DOI:** 10.3390/molecules15085196

**Published:** 2010-08-02

**Authors:** Miroslav Sisa, Susan L. Bonnet, Daneel Ferreira, Jan H. Van der Westhuizen

**Affiliations:** 1 Department of Chemistry, University of the Free State, Nelson Mandela Avenue, Bloemfontein, 9301, South Africa; E-Mails: sisa.miroslav@gmail.com (M.S.); bonnetsl@ufs.ac.za (S.L.B.); 2 Department of Pharmacognosy, Research Institute of Pharmaceutical Sciences, School of Pharmacy, The University of Mississippi, University, MS 38677, USA; E-Mail: dferreir@olemiss.edu (D.F.)

**Keywords:** photochemistry, photoinduction, phototransformation, UV radiation, flavonoids, polyphenols, tannins, chalcones, photooxygenation

## Abstract

Flavonoids and their photochemical transformations play an important role in biological processes in nature. Synthetic photochemistry allows access to molecules that cannot be obtained via more conventional methods. This review covers all published synthetic photochemical transformations of the different classes of flavonoids. It is first comprehensive review on the photochemistry of flavonoids.

## 1. Introduction

Flavonoids are polyphenolic compounds based on a C_15_ (C_6_C_3_C_6_) framework. They contain a chroman ring (C-ring) with a second aromatic ring (B-ring) at the C-2, C-3, or C-4 position. The heterocyclic six-membered C-ring is sometimes replaced by a five-membered ring (e.g., aurones) or the acyclic form (chalcones). The oxidation state of the C-ring is used to classify flavonoids into different categories, of which typical examples are flavan-3-ols, flavanones, flavones and flavonols. The term *flavonoid* can be ambiguous as it may refer either to the class of all C_6_C_3_C_6_ compounds, or its meaning may be restricted to 2-arylchromans with a carbonyl group at C-4 (C-ring) [1]. Flavonoids occur widely as glycosylated monomers or as flavan-3-ol oligomers (proanthocyanidins = condensed tannins). 

Epidemiological studies suggest that the regular consumption of flavonoids protects humans against diseases associated with oxidative stress such as Alzheimer’s disease [2], arteriosclerosis [3], cancer [4,5], and ageing [6]. The polyphenolic nature of flavonoids equates with ready oxidation and the formation of stable radicals and it is widely believed that flavonoids protect against free radical damage (caused by photolytically generated singlet oxygen and metabolic processes in living organisms) and act as antioxidants [7]. Other biological effects include improved blood flow [8], the inhibition of cholesterol absorption [9] and protection from damage by ultraviolet B radiation [10]. These have stimulated renewed interest in flavonoid synthesis and photochemical transformations that give access to molecules that are not available *via* conventional chemistry. The increasing use of flavonoids as food additives for health purposes has also contributed towards the growing interest in flavonoid photostability and photochemistry. Flavonoids are commercially important constituents of red wine [11], adhesives [12], and black tea [13].

The reason for the ubiquitous existence of flavonoid monomers and their oligomers as secondary metabolites in plants is controversial. Their polyphenolic nature allows complexation with proteins, as is evident in the widespread use of tannin extracts to tan leather, hence the name tannin [14]. This property renders protein in food indigestible and supports their putative anti-feeding role that provides protection against insect predation. Certain flavonoids are toxic to insects and other organisms. Bark that contains rotenoids is used by tribal communities to poison and harvest fish from rivers [15].

The light absorption properties of flavonoids and anthocyanidins in the visible ultraviolet light region are responsible for the colours associated with flowers and this plays an important role in pollination by insects and thus plant reproduction.

It has been shown that light is important in flavonoid biosynthesis [16,17], and that light is essential for anthocyanidin synthesis [18,19,20,21]. Flavonoids play important roles as development regulators and can regulate auxin transport *in vivo* [22]. Their role as antioxidants in plants [23], in stress protection [17] and in photoprotection [24] has been discussed. The influence of light on plant defence against pest and pathogens has been reviewed [25]. 

The postulate that flavonoids protect plants against ultraviolet light damage is supported not only by the fact that flavonoids absorb UV radiation and may act as sunscreens, but also by observations that exposure to UV radiation induces higher levels of flavonoids in plants. Caldwell [26] demonstrated a correlation between flavonoid content in plants and ambient UV conditions. Alpine plants at high altitudes and tropical plants from regions exposed to intense UV radiation have higher flavonoid content than plants from other regions. Plants exposed to sunlight have short internodes and smaller thicker leaves than plants that grow in shade [17].

It was observed that the biosynthesis of flavonoids with antioxidant properties (e.g., orthodihydroxy or catechol B-ring substitution) in plants is stimulated by UV light at the expense of flavonoids that are not considered as antioxidant (e.g., monohydroxy B-ring substitution) and flavonoids with good sunscreen properties (e.g., hydrocinnamic acid derivatives). This suggested that flavonoids’ photoprotection may also involve the removal of reactive oxygen species that form as a result of exposure to strong UV light.

Apart from the photochemical transformations that flavonoids may undergo due to their long daily exposure to sunlight, these compounds may also transfer or accept light energy to or from other molecules, *i.e.* act as sensitizers or quenchers.

A photochemical transformation requires excitation of an electron from a ground state orbital to an excited state orbital. This is usually achieved *via* the absorption of ultraviolet light (UV) by a chromophore. All flavonoids have aromatic chromophores, as indicated by UV absorptions in the 250 nm region of their UV spectra. These compounds may undergo π,π* excitation and react from π,π* excited states. Certain flavonoids contain carbonyl chromophores and absorb light in the 300 nm region. They may undergo n,π* excitation to react from n,π* excited states. Carbonyl chromophores that are conjugated with the aromatic ring (e.g., acetophenones and chalcones) absorb UV light in the 350 nm region. The n,π* and π,π* excited states of these compounds are almost degenerate and the state from which their reactions originates is sometimes controversial. Polyphenolic chalcones may absorb light in the visible region as is evident by their colours. Molecules that have no chromophores and cannot absorb light energy may be excited indirectly *via* sensitisation. This involves the transfer of mostly triplet energy.

The excited states may be in the triplet or singlet form. The triplet excited n,π* state [^3^(n,π*) state] is associated with radical reaction type products and the singlet excited π,π* state [^1^(π,π*) state] with ionic reaction type products. Solvent polarity is important and ionic type products are encouraged by polar solvents. Triplet excited states are formed indirectly from the initially formed singlet states *via* intersystem crossing [27]. Triplet excited states usually have much longer lifetimes than the corresponding singlet states, permitting photochemical transformations to compete more effectively with relaxation of the excited state to the photochemical inert ground state.

Much of the older photochemistry work in flavonoid chemistry was on compounds with unsubstituted aromatic rings and high yields were reported (see the review by Gupta *et al.* [28]). Polyphenolic flavonoids are more representative of naturally occurring molecules and have more interesting biological properties. These compounds generally afford lower yields and require special conditions to react due to deactivation by phenolic hydroxy and methoxy groups [29]. 

## 2. Flavone and Flavonol Photochemistry

Flavones and flavonols are characterized by fully unsaturated C-rings that connect the A and B-rings in a single conjugated system. They are generally photochemically inert as indicated by their reported use as photosensitisers, photoquenchers and ultraviolet absorption filters [30]. Their inertness prompted and allowed investigation into the potential of photochemically generated singlet oxygen to afford chemical transformations.

**Scheme 1:**
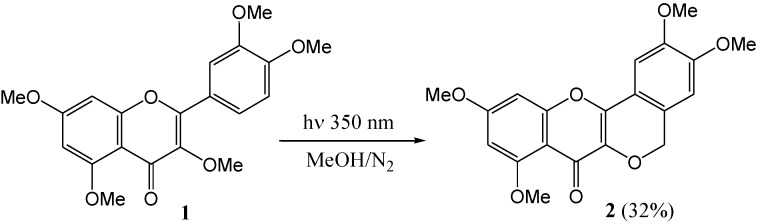
Photo-oxidative cyclization of quercetin pentamethyl ether.

**Figure 1:**
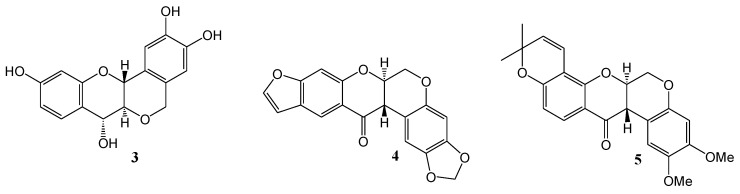
Structure of peltogynol and the naturally occurring rotenoids.

Waiss and Corse (1965) [31] investigated per-*O*-methylflavonols. Photoxidative cyclisation of quercetin penta-*O*-methyl ether (**1**) afforded the tetra-*O*-methyl ether of β-photomethylquercetin (**2**) in 32% yield in deoxygenated methanol under dry nitrogen (low pressure mercury lamps, 350 nm) (Scheme 1). This result led the authors to speculate that photochemistry was involved in the biosynthesis of peltogynol (**3**) and the rotenoids [e.g., dolichone (**4**) and α-toxicarol (**5**) (Figure 1], which co-occur with 2-methoxyisoflavonoids.

**Scheme 2:**
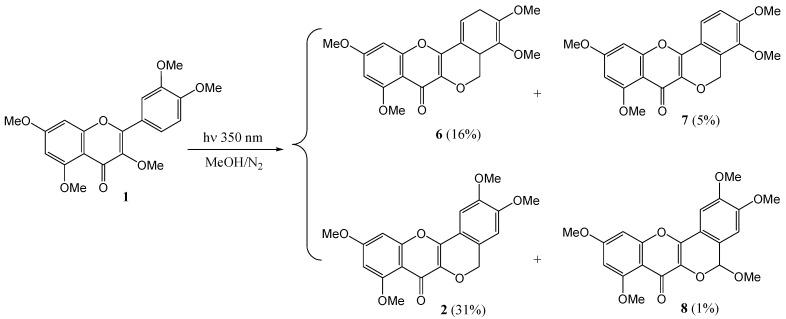
Photochemistry of quercetin pentamethyl ether.

**Figure 2:**
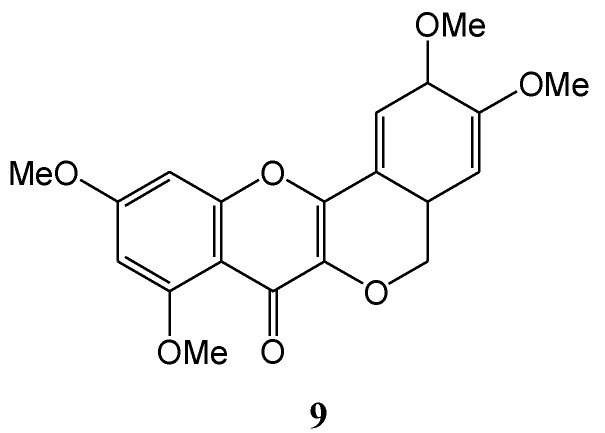
Structure of β-lumimethylquercetin.

In a subsequent paper Waiss and co-workers (1967) [32] described the isolation under similar conditions of β-photomethylquercetin (**2**, 31%), α-lumimethylquercetin (**6**, 16%), α-photomethyl-quercetin (**7**, 5%), and traces of methoxy β-photomethylquercetin (**8**, 1%) (Scheme 2). No β-lumi- methylquercetin (**9**, Figure 2) was detected. Repetition of the reaction in oxygen-free benzene yielded only photo-oxidised β-photomethylquercetin, and α-photomethylquercetin, at twice the rate of reaction. Rigorous efforts to exclude oxygen failed to yield α-and β-lumimethylquercetins. Yields in benzene were not reported. Failure to inhibit the reaction with triplet quenchers (anthracene or O_2_) and low intensity of phosphorescence as compared to fluorescence led to postulation of a ^1^(π,π*) primary reactive intermediate.

**Scheme 3:**
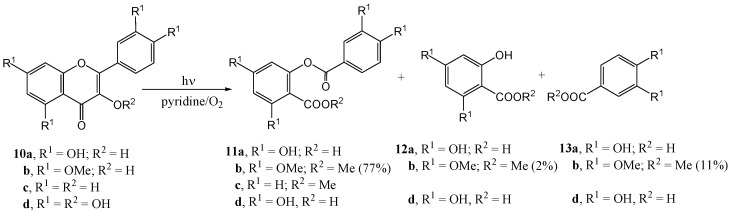
Photosensitized oxygenation of 3-hydroxyflavones.

**Scheme 4:**
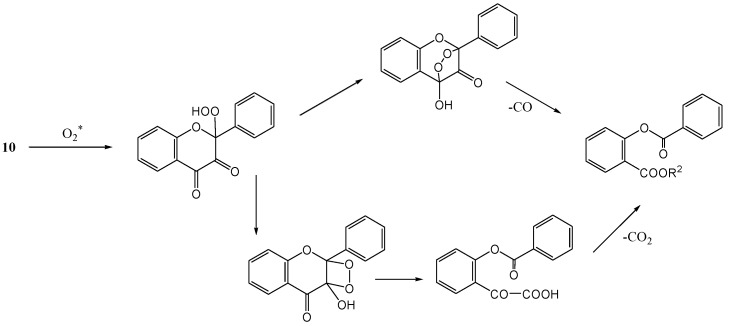
Suggested mechanism of photosensitized oxygenation of 3-hydroxyflavones.

Matsuura and Matsushima [33] studied flavonols where the 3-hydroxy group had not been methylated. Photolysis of quercetin 5,7,3',4'-tetra-*O*-methyl ether (**10b**) in pyridine in the presence of rose bengal under bubbling oxygen (300 W tungsten lamp) followed by diazomethane methylation of the reaction mixture afforded depside (**11b**, 77%), methyl 2-hydroxy-4,6-di-*O*-methylbenzoate (**12b**, 2%) and methyl 3,4-di-*O*-methylveratrate (**13b**, 11%) (Scheme 3). Enzymatic oxygenation also yielded depside and there seemed to be a resemblance between enzymatic and photosensitised oxygenation. Liberated carbon monoxide (31%) and carbon dioxide (17%) were determined. Similar results were obtained with 3-hydroxyflavone (**10c**). In a later publication Matsuura and co-workers [34] irradiated (100 W high-pressure lamp) free phenolic quercetin (**10d**) in methanol in the presence of rose bengal to get **12b**. In the absence of an oxygen sensitiser no reaction took place. No reaction was observed with quercetin 3,7,3',4'-tetra-*O*-methyl ether. It was suggested that the 3-hydroxy group was essential for photo-oxygenation. The mechanism for the reaction is given in Scheme 4.

**Scheme 5:**
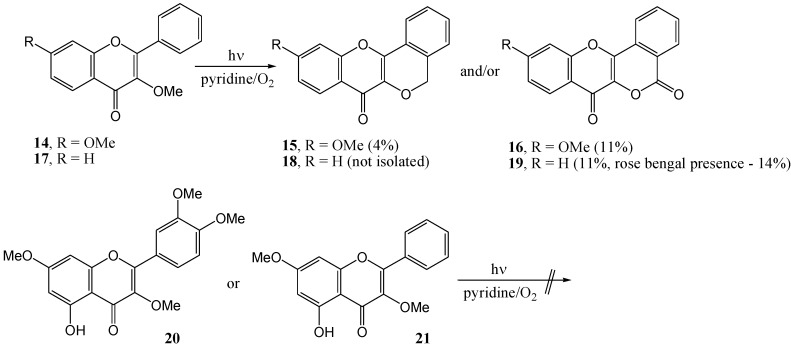
Photo-oxidative cyclization of 3-methoxyflavones.

Matsuura and Matsushima [35] postulated that, despite rigorous efforts by Waiss and co-workers [31,32] to work under oxygen-free conditions, oxygen must be involved in the transformation of intermediary lumimethylquercetin to photomethylquercetin. Photolysis of 3,7-dimethoxyflavone (**14**) under oxygen in pyridine (high-pressure mercury lamp with a Pyrex filter) yielded at least seven compounds. Only the photomethyl analogue (**15**, 4%) and the lactone (**16**, 11%) were isolated. 3-Methoxyflavanone (**17**) yielded only the lactone (**19**, also 11%) (Scheme 5). 

**Scheme 6:**
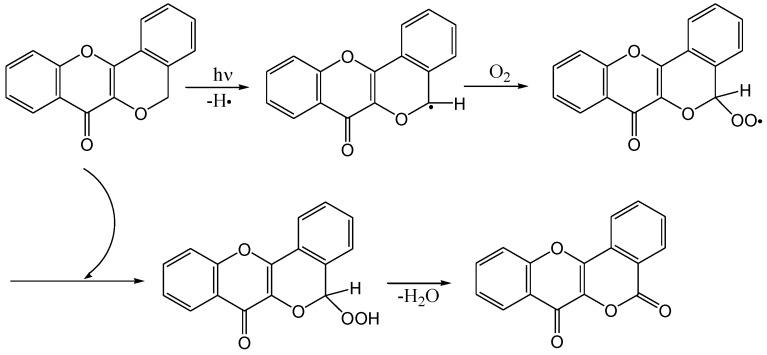
Mechanism of the formation of the lactone.

**Scheme 7:**
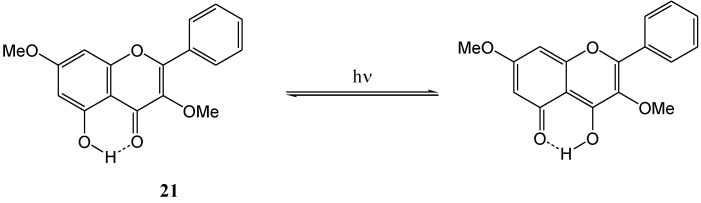
Photostabilization of *o*-hydroxybenzophenone.

The mechanism of the formation of the lactone is given in Scheme 6. Quercetin 3,7,3',4'-tetra-*O*-methyl ether (**20**) and 5-hydro-3,7-dimethoxyflavone (**21**) did not undergo photocyclisation and were recovered unchanged. This was attributed to hydrogen bonding between the 4-carbonyl and 5-hydroxyl group that interferes with the n,π* excitation of the carbonyl chromophore. This conclusion is supported by the fact that *o*‑hydroxybenzophenone does not undergo photoreduction and is used as a photostabiliser (Scheme 7). 

**Scheme 8:**
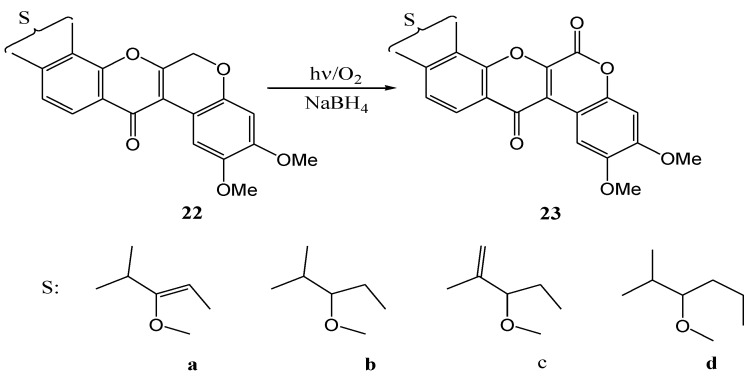
Photoinduced oxygenation of dehydrorotenones.

Suginome and co-workers [36] studied photo-oxygenation of dehydrorotenoids. Irradiation of dehydrorotenone (**22c**) with a 150 W high-pressure mercury lamp in a dioxane-ethanol mixture containing sodium borohydride yielded rotenonone (**23c**, 40%) (Scheme 8). In the absence of sodium borohydride no isolable product was formed. It was postulated that sodium borohydride converted intermediary hydroperoxides (the product of singlet oxygen attack on the methylene moiety) into hemiacetals that converted to the lactone carbonyl in the product. Sodium borohydride may also destroy free radical initiators and prevent unwanted side reactions. Table 1 gives yields obtained with analogues of dehydrorotenones **23a-d**. Rotenone and isorotenone were inert under the reaction conditions.

**Table 1:** Photoinduced oxygenation of dehydrorotenones.

Irradiated compound 22	Product 23 (time of irrad./h)	Yield (%) with NaBH_4_	Yield (%) without NaBH_4_
**a**	**a** (44)	40	trace
**b**	**b** (24)	9	trace
**c**	**c** (24)	10	trace
**d**	**d** (94)*	16	trace
Rotenone ( **22c**: 6a,12a-dihydro-)	( **23c**: 6a,12a-dihydro-)	0	0
Isorotenone ( **22a**: 6a,12a-dihydro-)	( **23a**: 6a,12a-dihydro-)	0	0

* benzene-dioxane (2:1) as the solvent.

**Scheme 9:**
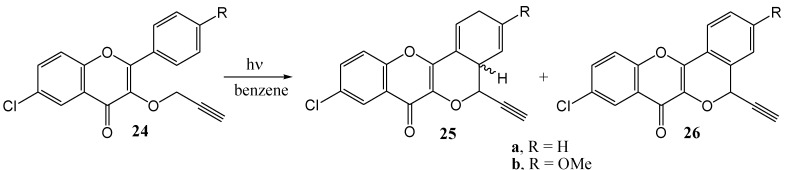
Phototransformations of 3-*O*-propargylflavonol.

Thakur and co-workers [37] investigated the photochemistry of 3-*O*-propargylflavonol (**24**). Irradiation with Pyrex filtered light from a 125 W Hg lamp in dry benzene furnished cyclized products **25a** (20%), **25b** (16%), **26a** (35%) and **26b** (30%), as shown in Scheme 9. 

**Scheme 10:**
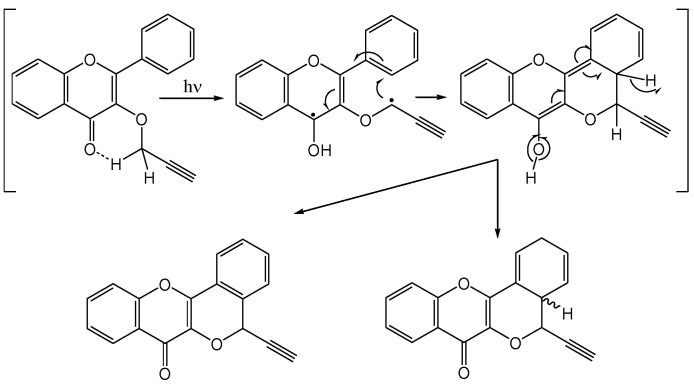
1,4-Hydrogen abstraction in propargylethers.

The mechanism was rationalised in terms of γ-hydrogen abstraction from the propargyl group by the excited carbonyl group followed by cyclisation of the 1,4-biradical (Scheme 10). 

**Scheme 11:**
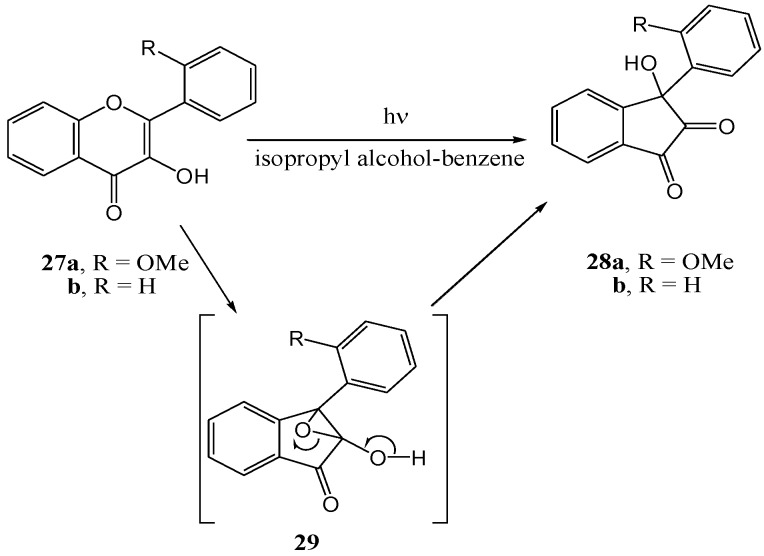
Photorearrangement of 3-hydroxyflavones.

Matsuura and co-workers [38] irradiated flavonol **27b** in isopropyl alcohol-benzene with a high pressure mercury lamp through Pyrex and obtained 3-aryl-3-hydroxy-1,2-inandione (**28b**) in almost quantitative yield. Irradiation of 2'-methoxyflavonol **27a** yielded **28a**. A 2,3-epoxy-2-hydroxy-1-inandone intermediate **29**, formed *via* a formal [_σ_2 + _π_2] cycloaddition, was postulated (Scheme 11).

**Scheme 12:**
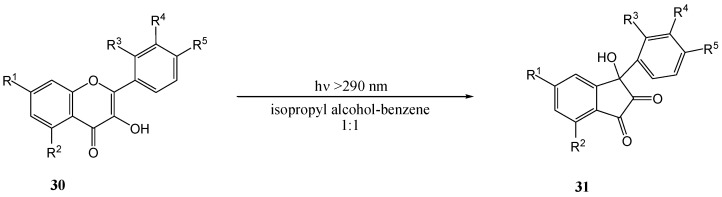
Photorearrangement of 3-hydroxyflavones to 3-aryl-3-hydroxy-1,2-indandiones.

**Scheme 13:**
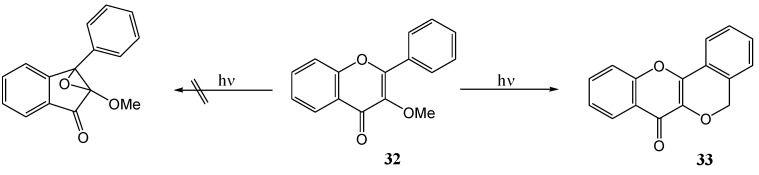
3-OMe-group prevention of the rearrangement to an inandione.

Subsequently Matsuura and co-workers [39] expanded the range of photolysed flavonols (Scheme 12). Table 2 includes previous results of Matsuura *et al*. [38] and the yields. Free phenolic quercetin (**30e**) resisted photo-transformation. This could be due to tautomerisation at the 4-CO and 5-OH (cf. photo-enolisation of *o-*hydroxyacetophenone [40,41]), internal quenching *via* the hydrogen bond between 4-CO and 5-OH, or deprotonation from the excited state (cf. resistance of 3-hydroxy-benzophenone to photoreduction [42]). Irradiation of the 3-*O*-methoxyflavonol **32** yielded a tetracyclic product **33**. The replacement of the 3-OH group (*i.e.* **30**) with a 3-OMe group (compound **32**) prevents the rearrangement to an inandione (compound **31**) (Scheme 13).

**Table 2:** Yields of indandiones obtained by photorearrangement of 3-hydroxyflavones.

30	R^1^	R^2^	R^3^	R^4^	R^5^	31	Yield (%)
**a**	H	H	OMe	H	J	**a**	Quant.
**b**	H	H	H	H	H	**b**	84
**c**	H	H	H	H	OMe	**c**	71
**d**	OMe	OMe	H	OMe	OMe	**d**	69
**e**	OH	OH	H	OH	OH	**e**	-

**Scheme 14:**
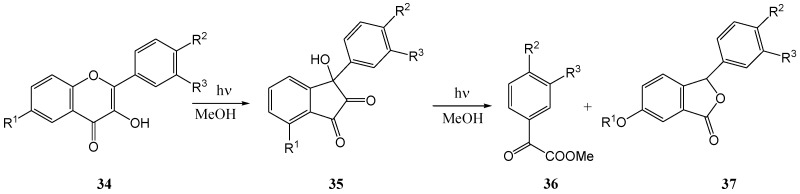
Photorearrangement of flavonols.

Yokoe and co-workers [43] irradiated flavonol **34a** in methanol with a high-pressure mercury lamp and obtained, in addition to the 3-aryl-3-hydroxy-1,2-inandione (**35a**, 8%) [38], a phthalide **37a** (0.7%) (Scheme 14). 

**Scheme 15:**
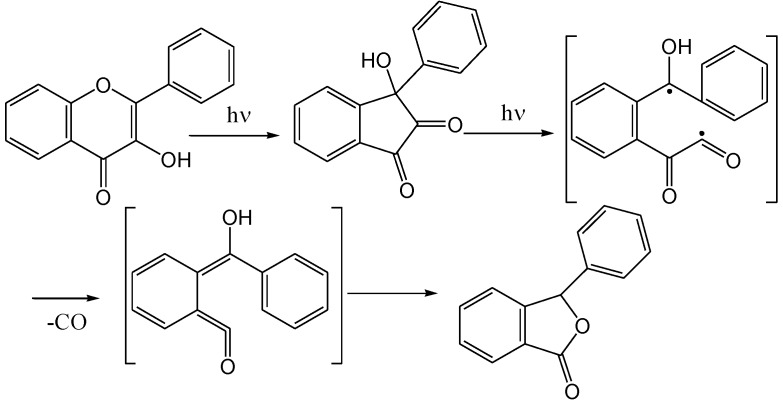
Mechanism of Norrish type I formation of phthalide.

The formation of phthalide **37a** was explained in terms of a photoinduced carbon monoxide loss from C-4 *via* a repeated Norrish type I process (α-fission) of the aliphatic carbonyl group (Scheme 15). The reaction was repeated with a range of substituted flavonols (Scheme 14 and Table 3) and in some cases an aromatic α-ketoacid, of type **36** was isolated. It was concluded that the formation of phthalides was, despite the poor yields, a general photoreaction of flavonols. In some cases a benzoic acid degradation product was also isolated. The presence of metal ions such as Cu^2+^, Ni^2+^, Fe^3+^, Co^2+^, and Be^2+^ prevented the photochemical rearrangement of flavonols. In contrast to these ions, Ca^2+^, Mg^2+^, and Hg^2+^ had no effects on the photochemical reactivities of flavonols. 

**Table 3:** Products obtained by the irradiation of flavonol in methanol.

	Yields (%)		
**34**	**35**	**36**	**37**
**a**, R^1^ = H, R^2^ = H, R^3^ = H	8		0.7
**b**, R^1^ = H, R^2 ^= Me, R^3^ = H	44		2
**c**, R^1^ = H, R^2^ = OMe, R^3^ = H		6	12
**d**, R^1^ = H, R^2^ = O-CH_2_-O = R^3^		14	6
**e**, R^1^ = Me, R^2^ = H, R^3^ = H	15		2
**f**, R^1^ = Me, R^2^ = Me, R^3^ = H			3
**g**, R^1^ = Me, R^2^ = OMe, R^3^ = H		1	0.2
**h**, R^1^ = Me, R^2^ = O-CH_2_-O = R^3^		4	

**Scheme 16:**
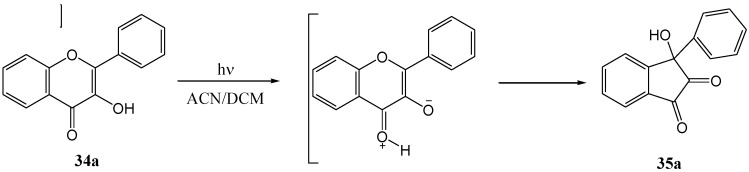
Irradiation of flavonol oxygen-free acetonitrile and dichloromehane solutions.

**Figure 3:**
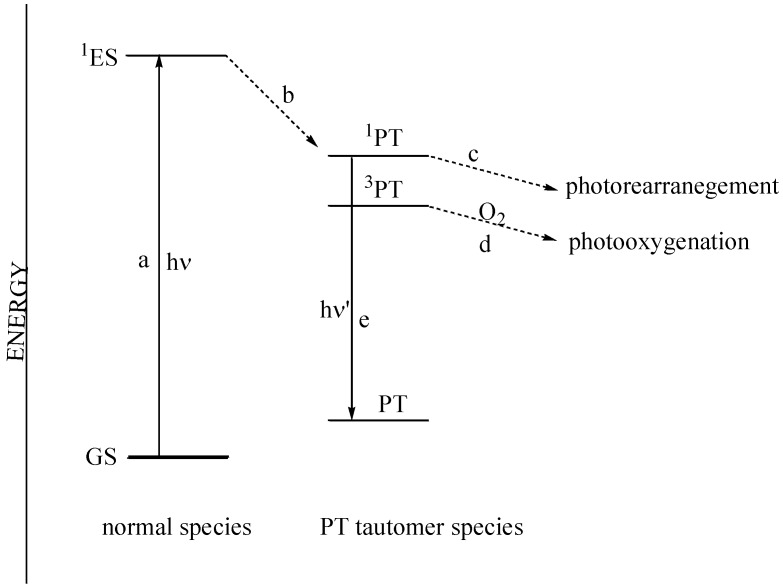
Proposed flavonol excited states: (GS ground state (singlet), ^1^ES excited state (singlet), PT ground state of phototautomer (singlet), its singlet excited state (^1^PT) and triplet excited state (^3^PT) a = light absorbtion; b = intersystem crossing; c and d = photochemical reaction; e = light energy emission).

Ficarra and co-workers [44] obtained only the indandione **35a** upon irradiation of flavonol **34a** in aerated or oxygen-free acetonitrile and dichloromehane solutions (high pressure mercury lamp with Bausch Lamb monochromator) (Scheme 16). No indication of the formation of photo-oxygenated products was found. This contrasts with the results of Yokoe and co-workers [43], where the oxygenated product dominated. They concluded that photo-rearrangement takes place from a singlet excited phototautomer (^1^PT) and photo-oxygenation from a triplet phototautomer (^3^PT). Alcoholic solvents (protic polar solvents) slow down rearrangements from (^1^PT) and inhibit photo-rearrangement at the expense of photo-oxygenation from (^3^PT). Aprotic polar solvents have the opposite effect and do not interfere with photo-rearrangement of (^1^PT). Their formation of only the oxygenated product in heptane and other non-polar solvents was explained by a rapid conversion of (^1^PT) to (^3^PT) in non-polar solvents. They thus concluded that molecular oxygen affects photochemical reactions of flavones in alcoholic (protic-polar) and hydrocarbon (non-polar) solutions and does not participate in acetonitrile and dichloromethane (aprotic-polar) (Figure 3).

**Scheme 17:**
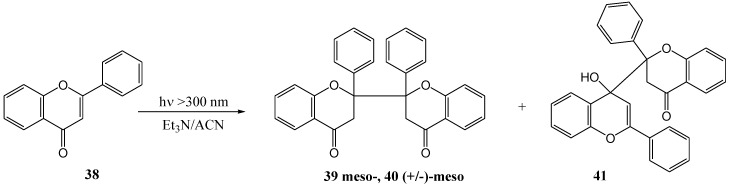
Photoinduced electron transfer reactions of flavone with amines.

Chen and co-workers [45] investigated the photoinduced electron transfer reactions of flavones with amines. Irradiation of flavone **38** with 0.1 M triethylamine in acetonitrile under argon at >300 nm yielded *meso*-2,2'-biflavanone **39**, (±)-*meso*-2,2'-biflavanone **40** and flavone [I-4,II-2]-flavanol **41** (Scheme 17). Different amines were used (TEA, DMBA, DMAE, DMA) in acetonitrile and benzene to afford yields, based on consumed flavone **38**, as shown in Table 4. These products resulted from radical addition of 4-ketyl **43** and/or its isomeric 1,2-ketyl anion to flavones, respectively (Scheme 19). Single electron transfer (SET) [46] is a well-known photoreaction between amines and α,β-unsaturated carbonyl compounds. The amine donates an electron to form an exciplex or a contact ion radical pair [47] (CIP) that undergoes hydrogen transfer to yield the radical responsible for dimerisation.

**Table 4:** Photoinduced reactions of flavone with amines.

Amine	Solvent	Irrad. Time/h	Conversion (%)	Products (yield, %)
TEA	ACN	14	100	**38** (38), **39** (14), **40** (25)
TEA	benzene	25	100	**38** (51), **40** (45)
DMBA	ACN	18	100	**38** (53), **40** (22)
DMAE	benzene	12	89	**38** (7), **39** (50), **40** (16)
DMA	ACN	14	100	**38** (41), **3** (35), **41** (18)
DMA	benzene	12	68	**38** (19), **39** (10), **40** (15), **41** (30)

**Scheme 18:**
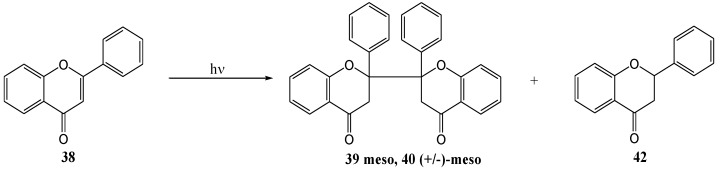
Photochemical synthesis of 2,2'-biflavanones from flavone.

Chen and co-workers [47] reinvestigated the photoinduced electron transfer reactions of flavone **38** with amines that was studied by his namesake in 1995. They used triethylamine or 2-(*N*,*N*-dimethylamino)ethanol at 250 and 300 nm in benzene, dichloromethane or acetonitrile, respectively, and isolated *meso*-2,2'-biflavone **39**, (+/-)-*meso*-2,2'-biflavone **40** and flavanone **42** (Scheme 18). 

**Scheme 19:**
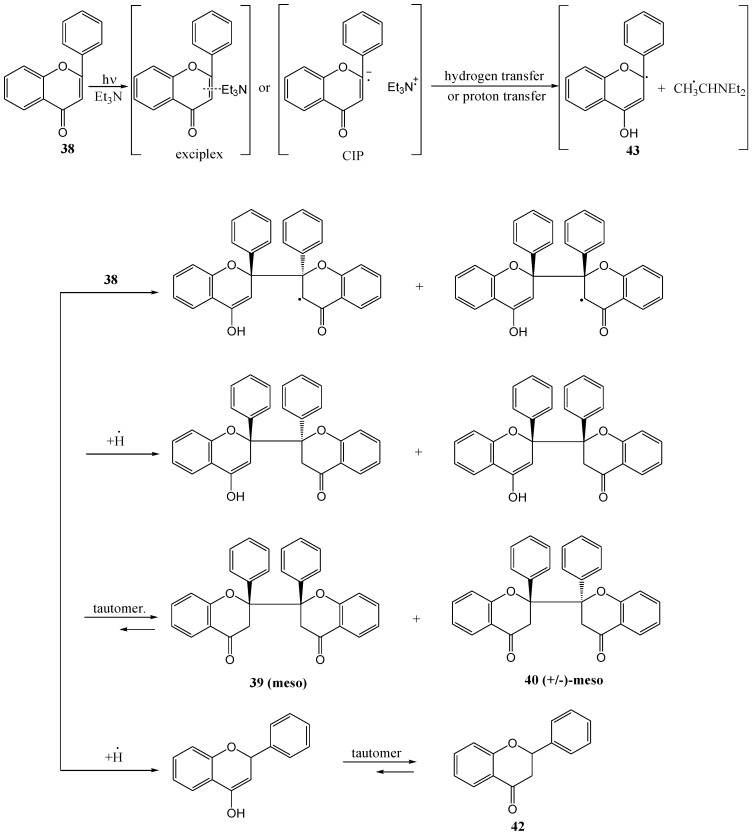
Proposed mechanism of photoinduced biflavanone formation with amines.

The mechanism of photolysis was proposed as shown in Scheme 19. The yields and the ratio between **39** and **40** depended on the molar ratio of flavone to amine, the type of amine, and the solvent and the light source (Sankyo 254 nm Germicidal lamp or 306 UV-B lamp) (Table 5). The combined yield of *meso*-**39** and (+/-)-*meso*-**40** varied beween 17.3 and 50.9%. They also isolated flavanone **42**, resulting from photo-reduction, in yields of between 8.9 and 16.9%, depending on the reaction conditions (Table 5). The absence of flavone [I-4,II-2]-flavan-4-ol **41** as described by Chen and co workers [45] above, was attributed to the use of light with a wavelength longer than 300 nm. 

**Table 5:** Yields and conditions of photoinduced biflavanone formation with amines.

Reaction Conditions	Conversion (%)	Yields (%)*^4^		
Molar ratios*^1^, Amines*^2^, Solvents, Irradiation Time, Irradiation Source*^3^		39	40	41
1/2, TEA, benzene, 25 hrs, A	75.0	22.0	22.3	13.0
1/4, TEA, ACN, 25 hrs, A	78.4	15.9	26.1	10.2
1/1, TEA, ACN, 14 hrs, A	88.5	12.2	9.7	11.6
1/2, TEA, ACN, 14 hrs, A	78.0	20.9	30.0	15.8
1/4, TEA, ACN, 14 hrs, A	85.4	16.7	14.6	14.4
1/10, TEA, ACN, 14 hrs, A	82.6	29.7	13.5	15.8
1/2, DMAE, benzene, 12 hrs, A	88.8	15.2	22.8	13.7
1/2, TEA, DCM, 16 hrs, A	71.1	17.4	7.9	16.9
1/2, TEA, benzene, 25 hrs, B	78.7	17.6	7.5	8.9
1/2, TEA, ACN, 14 hrs, B	75.8	12.5	4.8	11.9
1/2, DMAE, benzene, 14 hrs, A	79.1	9.6	19.3	11.2

*^1 ^Molar ratio of substrate to amine; *^2 ^Amine: TEA: triethylamine, DMAE: 2-(*N*,*N*-dimethylamino)ethanol. *^3 ^Irradiation Source: A (Sankyo 254 nm Germicidal Lamp), B (Sankyo 306 nm UV-B Lamp), *^4 ^Yield based on consumed flavone **1**.

Yokoe and co-workers [48] obtained similar results. Photolysis (450 W high pressure mercury lamp, Pyrex filter) of flavone **44** in a methanol-water solution in the presence of sodium sulphite yielded *meso*-2,2'-biflavanone **45** and (+/-)-*meso*-2,2'-biflavanone **46**.

**Scheme 20:**
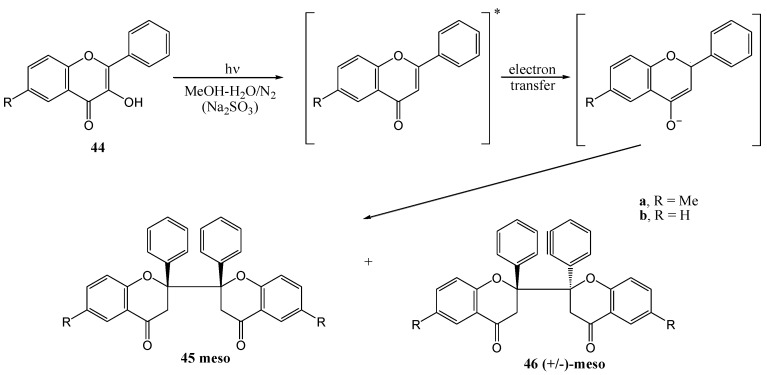
Photoinduced formation of biflavanones with sodium sulphite.

Schönberg and Khandelwahl [49] studied 1,2-cycloaddition to flavones. Irradiation of flavones **47a** and **47b**, respectively, -and diphenylacetylene in benzene with a Hanau quartz lamp in a quartz or Pyrex vessel yielded the corresponding cyclobutabenzopyrans **48a** and **48b**, respectively. Products **50**, **52** and **54**, respectively, were isolated upon irradiation of isoflavone **49**, 7,8-benzoflavone **51** and 1-thioflavone **53**, respectively. Yields were almost quantitative.

**Scheme 21:**
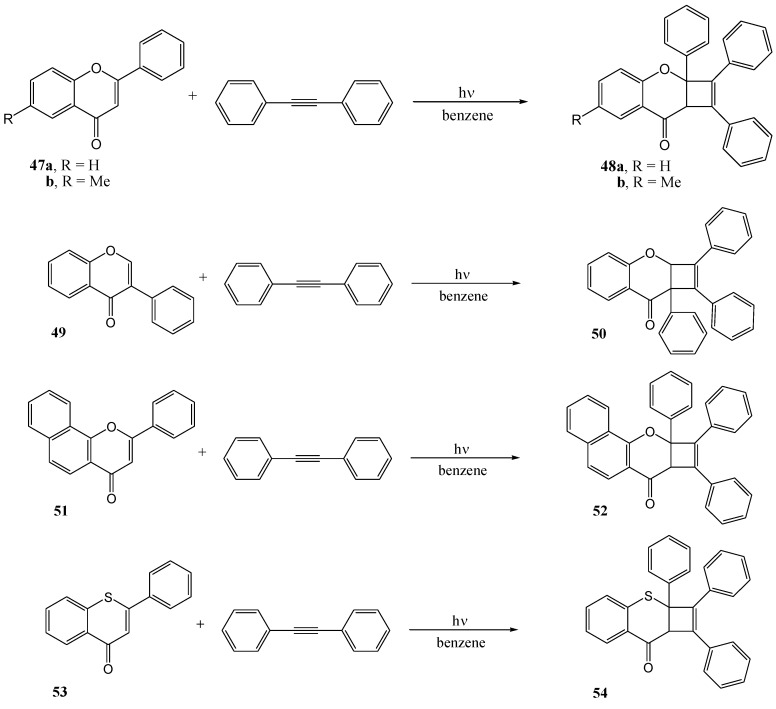
1,2-Cycloadition of diphenylacetylene and flavone.

**Scheme 22:**
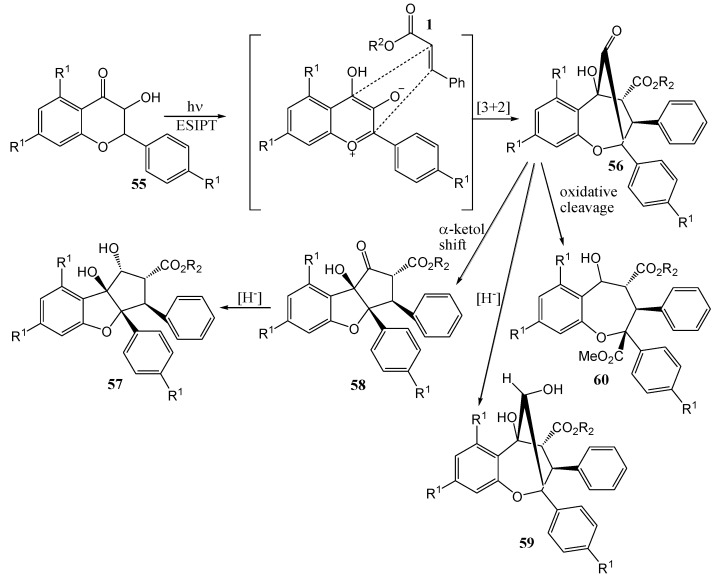
Photochemical synthesis of rocaglamide.

Gerard and co-workers [50] described 1,3-dipolar cycloaddition between methyl cinnamate (**55**) and flavonol **56** to obtain products **57** to **60** (rocaglamides) *via* photochemical generation of an oxidopyrylium species in acetonitrile with a Hanovia UV lamp uranium filter (Scheme 22). 

Gerard and co-workers [51] repeated the reaction in the presence of chiral Brønsted acids to obtain chiral rocaglamide derivatives. R^1^ and R^2^ are identical or different and selected from the group consisting of hydrogen, halogen, hydroxyl, alkoxy, aryloxy, heteoaryloxy, thioalkyl, thioaryl, and next variety of protecting groups.

Bhatacharyya and co-workers [52] studied the photophysics of flavones. They concluded that flavone almost instantaneously forms a triplet state with a 90% intersystem crossing (ISC) yield after absorption of UV light. Polar solvents enhanced yields indicating a π,π*-character for the lowest triplet excited state. The flavone’s triplet is quenched by several typical triplet quenchers like hydrogen donors [53], including amines [54].

**Scheme 23:**
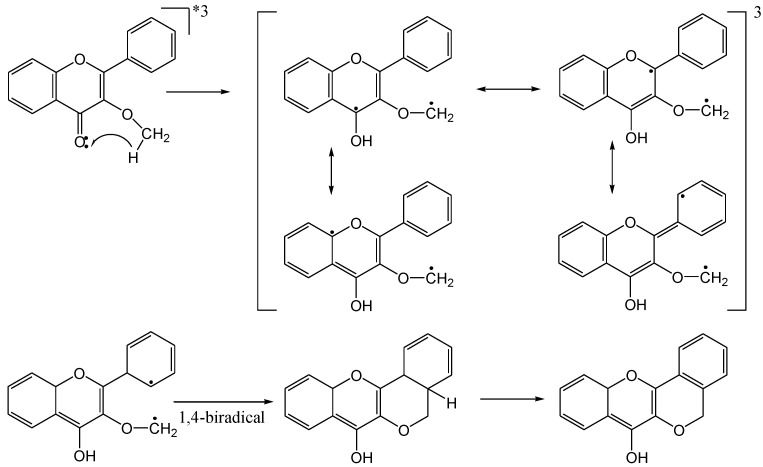
Mechanism of photocyclisation of the 3-methoxyflavone.

Christoff and co-workers [55] investigated the photophysics of 3-methoxy- and 7-methoxyflavone. A 7-methoxy substituent increases the π,π*-character of the excited state further but does not change the energy level or known flavone deactivation pathways. In contrast, a 3-methoxy substituent leads to a strong geometric constraint which interferes with planar π-orbital conjugation between the carbonyl group and the aromatic ring. This reduces the π,π*-character and increases the n,π*-character and the triplet state energy. The methoxy group becomes an intramolecular hydrogen donor to the excited carbonyl n,π*-triplet. The spectroscopic properties of the transient species are compatible with a 1,4-biradical structure. Conventional photolysis indicates that the biradical is transformed into an ethereal ethylene group. The mechanism for the photocyclisation is given in Scheme 23.

## 3. Chalcone and Flavanone Photochemistry

**Scheme 24:**
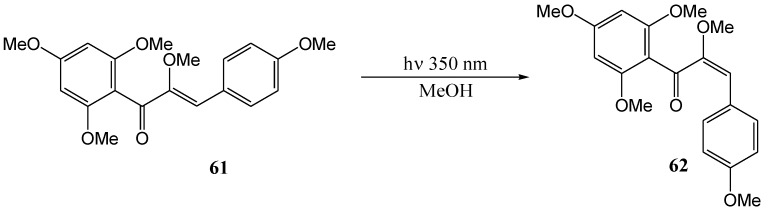
*Trans*-*cis* – photoinduced isomerisation of α-methoxychalcone.

Chalcones and α-hydroxychalcones are key intermediates in flavonoid synthesis and biosynthesis and the photochemistry of these compounds has attracted much early interest. Of major interest is *cis-trans* isomerisation of the olefinic bond and reversible interconversion of 2'-hydroxychalcones and flavanes. Lutz and Jordan [56] obtained a photo-stationary mixture of 74% *cis*- and 26% *trans*-chalcone upon leaving the chalcone in benzene in sunlight. The *trans*-isomer absorbs light more efficiently and is depleted compared to the *cis*-isomer under the reversible reaction conditions. Volsteedt and co-workers (1973) [57] isomerised *trans*-α,2',4,4',6'-pentamethoxychalcone (**61**) to the *cis* form **62** in methanol at 350 nm (Scheme 24).

**Scheme 25:**
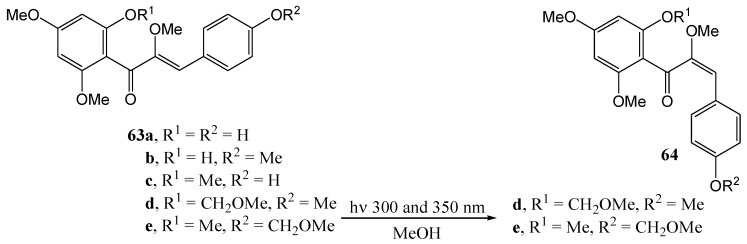
*Trans*-*cis* – photoinduced isomerisation of α-methoxychalcones.

**Figure 4:**
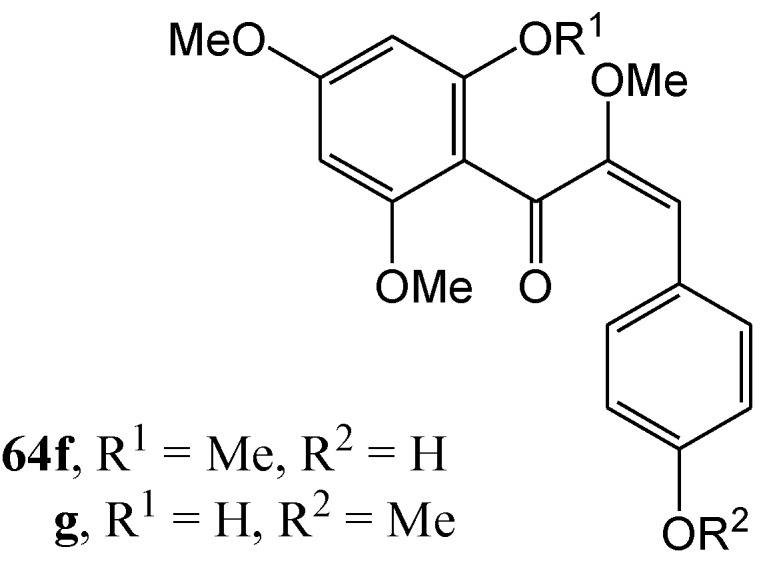
Structures of photochemical relatively stable 2'-hydroxy-α-methoxy-*cis*-chalcones

Ferreira and Roux [58] found that a *trans*-chalcone with a free hydroxy group (e.g., 2'-hydroxy-4,4',6'-trimethoxy- and 4-hydroxy-2',4',6'-trimethoxy*-trans*-chalcone **63a**, **63b** and **63c**) did not undergo *cis*-*trans* isomerisation in methanol at 300 or 350 nm. Their *O*-methoxymethyl derivatives **63d** and **63e** did undergo the desired isomerisations to a mixture of 60% *trans* (compound **64d**) and 40% *cis* (compound **64e**) (Scheme 25). The products were, however, metastable and spontaneously reverted to the *trans* isomer (half-life of about six months). Removal of the *O*-methoxymethyl protecting group of the *cis* chalcones **64d** and **64e** with acid, yielded the corresponding *trans* isomers **63b** and **63c**. In contrast 2'-hydroxy-α-methoxy-*cis*-chalcones **64f** and **64g** are relatively stable (Figure 4). 

**Scheme 26:**
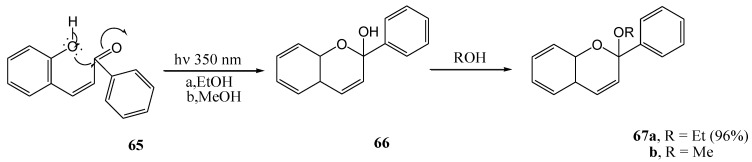
The photochemistry of 2''-hydroxychalcone.

Dewar and Sutherland [59] transformed 2''-hydroxychalcone **65** into 2-ethoxy- or 2-methoxyflav-3-ene (compounds **66a** or **b**) (96%), and trace amounts of flavones (1%), upon irradiation at 350 nm in ethanol or methanol (Scheme 26). The product (**67a** or **b**) was smoothly converted to a flavylium salt with acid.

**Scheme 27:**
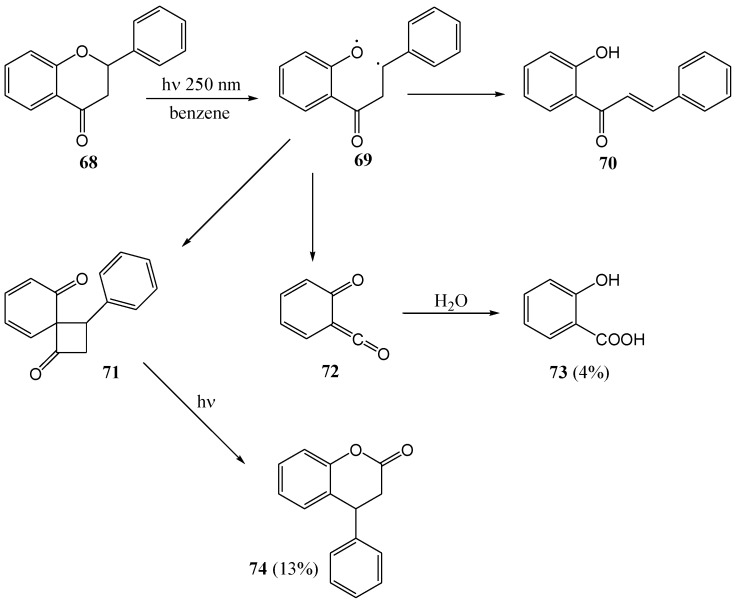
Photochemistry of flavanone.

Mack and Pinhey [60] transformed flavanone **68**, *via* fragmentation of the heterocyclic benzylic O-C bond, into 2'-hydroxychalcone **70** (20%) in benzene at 250 nm. They also isolated salicylic acid (**73**, 4%) *via* the subsequent fragmentation of the C2-C3 bond to form a ketene **72** that can be trapped with water or methanol. The 4-phenyldihydrocoumarin (**74**, 13%) was formed *via* attack of the aromatic A-ring on the benzylic radical intermediate **69 **to yield the intermediate dienone **71** (Scheme 27). This reaction is related to the photo-Fries rearrangement of benzylic ethers.

**Scheme 28:**
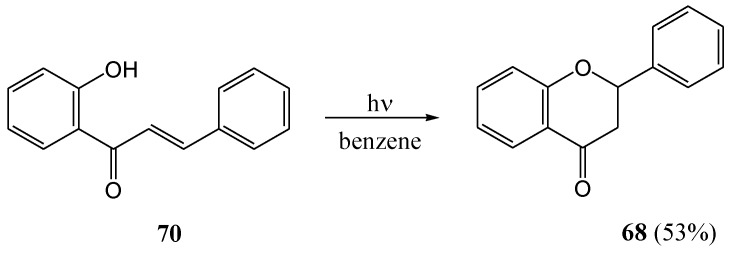
Photochemical transformation of 2'-hydroxychalcone to flavanone.

Stermitz and co-workers [61] transformed 2'-hydroxychalcone **70** to flavanone **68** (53%) upon irradiation with a Hanovia 450 W lamp with a Pyrex filter in benzene. It is known that the pKa values of phenols increase in the excited state [62] and, thus, the reaction could have been acid self-catalyzed (Scheme 28).

**Scheme 29:**
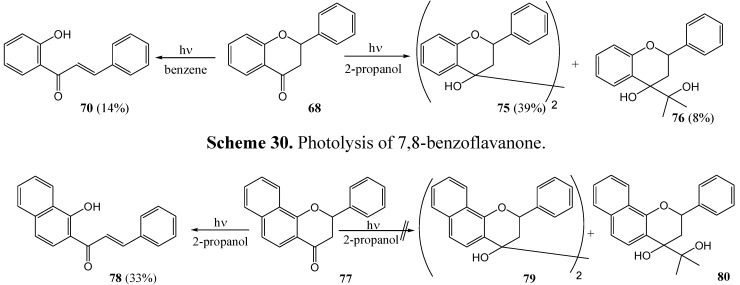
Photolysis of flavanone in 2-propanol and benzene.

**Scheme 30:**
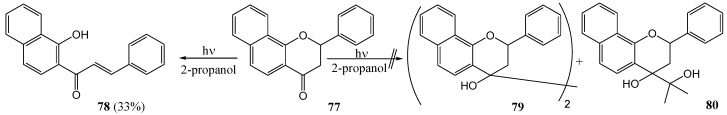
Photolysis of 7,8-benzoflavanone.

Matsushima and co-workers [63] photolysed (100 W high pressure mercury lamp) flavanone **68** in benzene and obtained 2'-hydroxychalcone **70** (14%) *via* cleavage of the pyrone ring. Repetition of the reaction in 2-propanol gave the pinacols **75** (39%) and solvent adducts **76** (8%) (Scheme 29). Photolysis of 7,8-benzoflavanone **77** in 2-propanol gave no coupling products **79** and **80**, but rather the ring-cleaved chalcone **78** (33%) (Scheme 30). The lack of photo-reduction (coupling products) suggested that the lowest triplet state of 7,8-benzoflavanone was ^3^(π,π*) and that of the flavanones that had undergone photo-reduction to pinacols were ^3^(n,π*). Methoxylated flavanones are assumed to have considerable π,π* character in their lowest triplet state. This is supported by conversion of 4'-methoxyflavan to 2'-hydroxy-4-methoxyflavanone in yields of 69% in benzene (compared to the 14% conversion of flavanone), 66% in pyridine, 48% in acetonitrile and 31% in carbon tetrachloride.

**Scheme 31:**
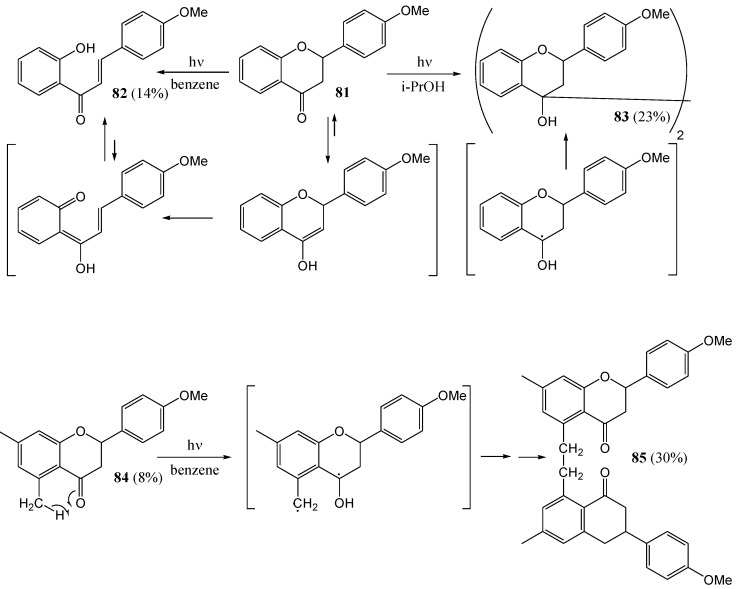
Photochemistry of 4'-methoxy- and 5,7-dimethyl-4'-methoxyflavanone.

Nakashima and co-workers [64] investigated the photochemistry of 4′-methoxyflavanone (**81**). They obtained chalcone **82** (14%), and a mixture of pinacols **83** (23%) upon irradiation of **81** with a high pressure Pyrex lamp in benzene or 2-propanol. 5,7,4'-Trimethoxyflavanone was inert in benzene and gave a complex mixture of at least six products that was not identified. 5,7-Dimethyl-4'-methoxyflavanone (**84**) yielded bis-flavanone **85**, probably *via* intramolecular hydrogen abstraction by an n,π*-excited carbonyl from the 5-methyl group (Scheme 31).

**Scheme 32:**
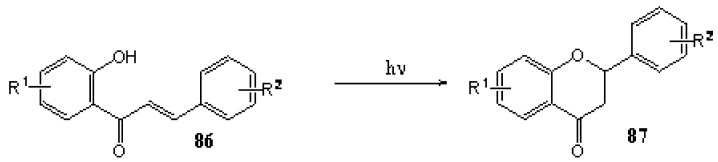
Photocyclization of 2'-hydroxychalcones to flavanones.

**Scheme 33:**
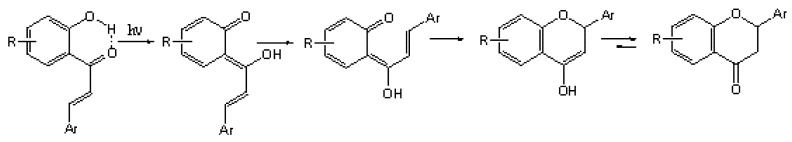
Mechanism of photocyclization of 2'-hydroxychalcone to flavanone.

Matsushima and Kageyama [65] studied the influence of the solvent and wavelength on the reactivity, reaction rate, and yield of photocyclisation of a series of 2'-hydroxychalcones **86a-e** to flavanones **87a-e** (Scheme 32, Table 6). They also investigated the effect of triplet quenchers and radical scavengers. All the chalcones were inert in chloroform and *t*-butyl alcohol and all were reactive in benzene, ethyl acetate, and 1,4-dioxane (Table 7). Light of a shorter wavelength gave slower reaction rates and more side reactions. The best results were obtained with wavelengths above 365 nm (Table 8). Rates were highest in ethyl acetate and dioxane and low in benzene. The yields were the highest in ethyl acetate [e.g., 86% conversion of **86b**] (Table 9). Triplet quenchers and radical scavengers had no effect, suggesting that photocyclisation of 2'-hydroxychalcones is an ionic or polar reaction. Solvent effects are ambiguous, but it seems that polar aprotic (basic) solvents gave the best results, supporting the polar mechanism. Photo-enolization of 3-chromanones as described by Padwa *et al* [66], followed by *cis*-*trans* isomerisation that may require a second photon, seems to best explain the experimental results (Scheme 33). 

**Table 6:** Photocyclization of 2'-hydroxychalcones to flavanones.

2'-Hydroxychalcone	R^1^	R^2^	Flavanone 87	R^1^	R^2^
86					
**a**	H	H	**a**	H	H
**b**	H	2-OMe	**b**	H	2'-OMe
**c**	4'-OMe	H	**c**	7-OMe	H
**d**	6'-OMe	H	**d**	5-OMe	H
**e**	3',4'-Benzo	H	**e**	7,8-Benzo	H

**Table 7:** Solvent effects on the photocyclization of 2'-hydroxychalcones to flavanones.

2'-Hydroxychalcone	Benzene	CCl_4_	CHCl_3_	Et_2_O	EtOAc	1,4-dioxane	ACN	EtOH	*t*-BuOH
86									
**a**	**87a**	**87a**	-	**87a**	**87a**	**87a**	**87a**	**87a**	-
**b**	**87b**	-	-	**87b**	**87b**	**87b**	**87b**	**87b**	-
**c**	**87c**	-	-	**87c**	**87c**	**87c**	-	-	-
**d**	**87d**	-	-	**87d**	**87d**	**87d**	-	-	-
**e**	**87e**	-	-		**87e**	**87e**	-	-	-

**Table 8:** Effect of the solvent on the relative rates of photochemical flavanone formation from 2'-hydroxychalcones.

2'-Hydroxychalcone 86	Consumption of 2'-hydroxychalcone (%) in		
	Benzene	1,4-dioxane	EtOAc
**a**	8.2	94	95
**b**	10	97	91
**c**	14	35	63
**d**	30	30	84
**e**	7.4	1.4	1.3

**Table 9:** Effect of irradiation time on the yield of photochemical flavanone **87b** formation from 2'-hydroxychalcones.

Solvent	Irradiation time/h	86b (mM Recovered)*^a^*	87b (mM Formed)	Yield of 87b (%)*^b^*
Benzene	5	0.77	0.17	74
	10	0.66	0.24	71
	15	0.55	0.34	69
	20	0.52	0.40	83
1,4-dioxane	1	0.79	0.17	82
	2	0.57	0.48	100
	3	0.31	0.55	79
	4	0.06	0.35	58
EtOAc	2	0.57	0.45	100
	3	0.32	0.62	91
	4.5	0.23	0.65	84
	6	0.14	0.86	100

*^a^* Initial concentration of **86b** was 1 mM; ^b^The yield of **87b** was based on the consumed amount of **86b**.

**Scheme 34:**
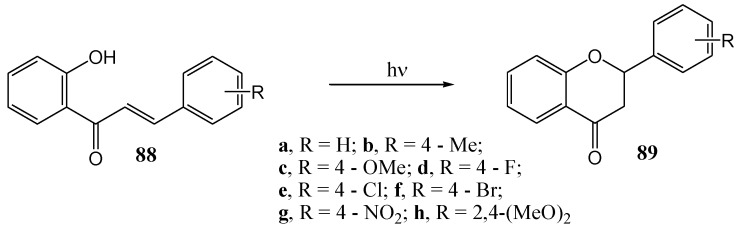
Photochemical cyclization of 2'-hydroxychalcones.

Matsushima and Kageyama [67] investigated the scope and mechanism of the photocyclisation of 2'-hydroxychalcones and photolysed a series of B-ring mono-substituted derivatives (Scheme 34). They concluded that visible light (405 nm) gives the best results *via* selective cyclisation, avoiding secondary reactions of the resulting flavanone such as hydrogen abstraction from the solvent or equilibration of the flavanone and chalcone. Methoxy and phenyl substituents on the B-ring enhanced the reaction rate while halogen atoms and a nitro group had little effect (Table 10). Solvent effects on the consumption rate of **88a** are given in Table 11. Reaction rates are the highest in aprotic polar solvents, low in non-polar solvents and extremely low in hydroxylic solvents that interfere with intramolecular hydrogen bonding (except in *t*-butyl alcohol). Ethyl acetate gave superior results compared to benzene and THF. Free radical inhibitors (2,6-di-*t*-butylphenol, nitrosobenzene, or acrylonitrile) did not suppress the reaction, suggesting a non-radical mechanism as supported by increased reaction rates in polar solvents. Triplet quenchers ferrocene, cyclohexa-1,3-diene, anthracene, phenanthrene, or acenaphthylene had no effect, suggesting an excited singlet or very short-lived triplet state. Quantum yields were low. In π,π* excited states phenolic groups become more acidic and carbonyl groups more basic [68,69,70] suggesting a charge transfer mechanism. 6-Methoxy-, 7-methoxy-, 4'-methoxy, 4'-carbomethoxy-, and 2-methyl-7-methoxyflavanones were also converted to the corresponding 2'-hydroxychalcones (no yields were reported).

**Table 10:** Photocyclization of 2'-hydroxychalcones with visible light.

Compound^a^		Solvent	Irrad. time (h)	Consumption of 88 (mM)	Formation of 89 (mM)	(89)/-(88)*^b^*
88	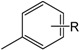					
**a**	Phenyl	EtOAc	10	0.79	0.72	0.91
		Benzene	10	0.24	0.12	0.50
		THF	5	0.30	0.25	0.83
**b**	4-Tolyl	EtOAc	10	0.67	0.64	0.93
		Benzene	10	0.18	0.14	0.77
		THF	5	0.34	0.22	0.63
**c**	4-Methoxy-phenyl	EtOAc	10	1.0	0.93	0.93
		Benzene	10	0.40	0.33	0.83
		THF	5	0..34	0.32	0.94
**d**	4-Fluorophenyl	EtOAc	5	0.59	*c*	
**e**	4-Chlorophenyl	EtOAc	10	0.93	0.82	0.88
		Benzene	10	0.17	0.18	1.0
		THF	5	0.49	0.37	0.75
**f**	4-Bromophenyl	EtOAc	10	1.0	0.74	0.74
		Benzene	10	0.17	0.16	0.94
		THF	5	0.24	0.18	0.75
**g**	4-Nitrophenyl	EtOAc	5	0.84	*c*	
**h**	2,4-Dimethoxy-phenyl	EtOAc	10	1.0	0.96	0.96
		Benzene	10	0.62	0.50	0.81
		THF	10	0.77	0.70	0.91
**i**	α-Naphthyl	EtOAc	10	1.0	0.94	0.94
		Benzene	10	0.45	0.32	0.71
		THF	10	0.65	0.61	0.94
**j**	Styryl*^d^*	EtOAc	10	0.45	0.31	0.69
		Benzene	10	0.15	0.06	0.40
		THF	10	0.15	0.09	0.60

^a ^Initial concentration of each chalcone was 1 nM. ^b ^Yield of flavone **89** based on the consumption of chalcone **88**. ^c^ Not determined. ^d^ 1-(2-Hydroxyphenyl)-5-phenylpenta-2,4-dien-1-one for comparison, although not a chalcone.

**Table 11:** Solvent effects on the consumption rate of 2'-hydroxychalcone **88a**.

Solvent^a^	Irrad. time (h)	Consumption (%)	Rate (% h^-1*b*^)
EtOAc	0.5	7.0	14
Dioxane*^c^*	2	14	7.0
Dioxane-H_2_O (95:5)	20	12	0.6
Dioxane-D_2_O (95:5)	20	13	0.65
THF *^c^*	2	11	5.4
THF-H_2_O (90:10)	5	6.4	1.3
THF-H_2_O (90:10)	5	7.0	1.4
*t*-Butyl alcohol	2	4.7	2.4
DMF	5	11	2.2
Cyclohexane	3	6.1	2.0
ACN	5	8.7	1.7
ACN-H_2_O (95:5)	50	0.0	0.0
ACN-H_2_O (40:60)	50	6.9	0.1
Benzene	14	8.1	0.6
Tetrachloromethane	40	11	0.3
EtOH	90	9.8	0.1
MeOH	90	7.7	0.1
Chloroform	40	0.0	0.0

*^a^* Initial concentration of **88a** was 1 mM, on visible irradiation. Irradiation times were controlled so that conversions were close to or below 10%. *^b^* Relative rate for the consumption of **88a**. Solvent effects on the absorption at λ ≥ 400 nm were nil, hence the rate can be regarded as the relative quantum yield. *^c^* Distilled over fresh sodium.

**Scheme 35:**
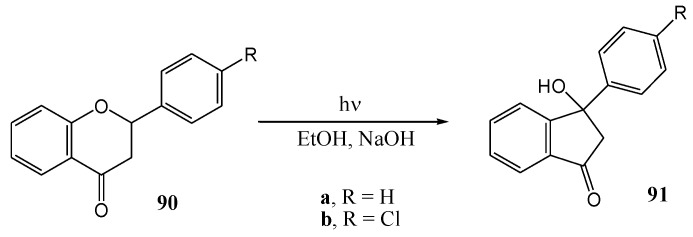
Photochemistry of flavanone and 4'-chloroflavanone in alkaline medium.

**Scheme 36:**
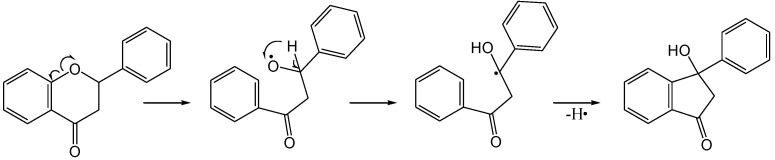
Proposed mechanism of photochemical rearrangement of flavanone in alkaline medium.

Jain [71] investigated the photochemistry of flavanones in alkaline medium. Flavanone **90a** and 4-chloroflavanone **90b** rearranged in alkaline medium (2 mL 10% NaOH in 2 mL ethanol) upon irradiation with UV of an unspecified source to the corresponding products **91a** and **91b** (Scheme 35), but no yields were reported. A concerted mechanism was proposed (Scheme 36). 

**Scheme 37:**
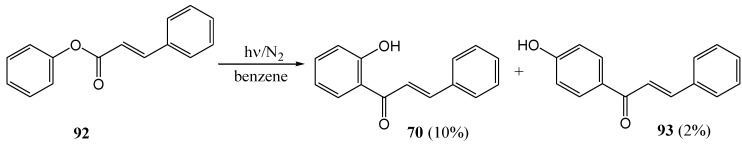
The photochemical Fries rearrangement of phenyl cinnamate to 2'-hydroxy- and 4'-hydroxychalcone.

Photochemical Fries rearrangement of phenyl cinnamates represents a convenient method to prepare 2'-hydroxychalcones. Obara and Takahashi [72] irradiated phenyl cinnamate (**92**) in benzene under nitrogen with a high-pressure 450 W mercury arc and obtained 2'-hydroxychalcone **70** (10%) and 4'-hydroxychalcone **93** (2%) (Scheme 37). 

**Scheme 38:**
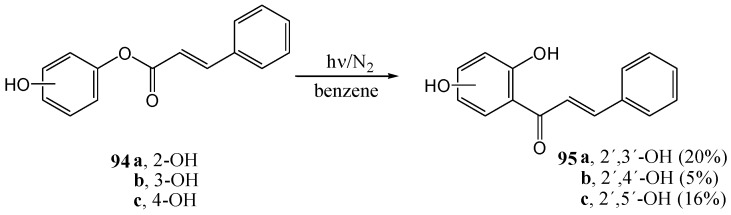
The photochemical Fries rearrangement of hydroxyphenyl cinnamate derivatives.

Obara and co-workers [73] repeated the reaction using a high-pressure 100 W mercury arc with 2-, 3-, and 4-hydroxyphenyl cinnamates **94a-c** and obtained the corresponding 2',3'-, 2',4'-, and 2',5'-dihydroxychalcones **95a-c** in yields of 20, 5 and 16%, respectively (Scheme 38). 

**Scheme 39:**
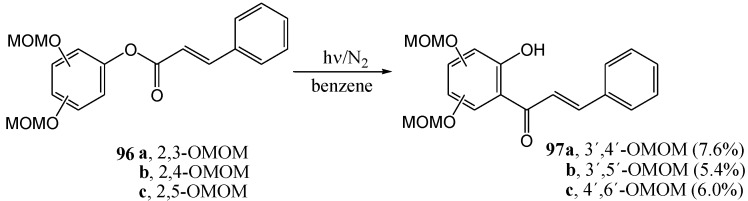
The photochemical Fries rearrangement of *O*-methoxymethyl protected dihydroxyphenyl cinnamates to the corresponding hydroxychalcones.

Onodera and Obara [74] extended the work to *O*-methoxymethyl-protected dihydroxyphenyl cinnamates **96a-c** and obtained the corresponding hydroxy-bis(*O*-methoxymethyl)-chalcones **97a-c** (Scheme 39). 

**Scheme 40:**
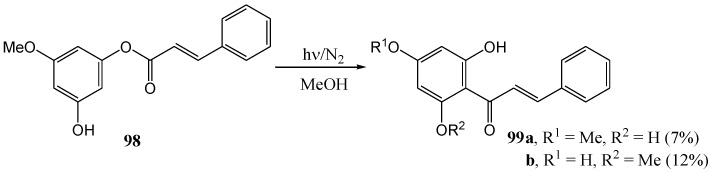
The photochemical Fries rearrangement of 3'-hydroxy-5'-methoxycinnamate into 2',6'-dihydroxy-4'-methoxychalcone.

Bhatia and Kagan [75] transformed cinnamate **98** into 2',6'-dihydroxy-4'-methoxychalcone (**99a**, 7%) and 2',4'-dihydroxy-6'-methoxychalcone (**99b**, 12%) by irradiation in methanol at 250 nm (Scheme 40).

**Scheme 41:**
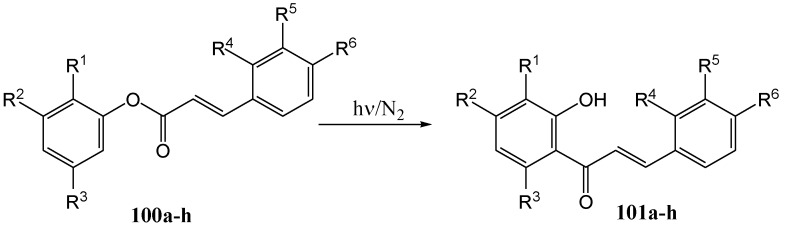
The photochemical synthesis of 2'-hydroxychalcones from phenyl cinnamates.

Ramakrishnan and Kagan [76] studied the photo-Fries reaction with the view of obtaining chalcones with the complex substitution patterns found in plants. A variety of chalcone derivatives were irradiated at 254 nm in different solvents, as given in Scheme 41 and Table 12. 

**Table 12:** R^1^ - R^6^ substituents on structure (**100**) and (**101**).

Substituent on structure 100 and 101	Solvent	Irradiation time (h)
**a**, R^1^ = R^2^ = R^3^ = R^4^ = R^5^ = R^6^ = H	MeOH or chloroform	20
**b**, R^1^ = OMe, R^2^ = R^3^ = R^4^ = R^5^ = R^6^ = H	chloroform	40
**c**, R^2^ = OH, R^1^ = R^3^ = R^4^ = R^5^ = R^6^ = H	EtOH	45
**d**, R^3^ = OH, R^1^ = R^2^ = R^4^ = R^5^ = R^6^ = H )*		
**e**, R^2^ = R^3^ = OH, R^1^ = R^4^ = R^5^ = R^6^ = H	MeOH	22
**f**, R^2^ = R^3^ = R^4^ = OH, R^1^ = R^5^ = R^6^ = H	MeOH	16
**g**, R^2^ = R^3^ = R^6^ = OH, R^1^ = R^4^ = R^5^ = H	MeOH	22
**h**, R^2^ = R^3^ = OH, R^6^ = OMe, R^1^ = R^4^ = R^5^ = H	MeOH	22
**i**, R^2^ = R^3^ = R^5^ = R^6^ = OH, R^1^ = R^4^ = H	MeOH	36
**j**, R^2^ = R^3^ = R^6^ = OH, R^5^ = OMe, R^1^ = R^4^ = H	MeOH	17
**k**, R^2^ = R^3^ = OH, R^5^ = R^6^ = OMe, R^1^ = R^4^ = H	MeOH	36

* **101d** obtained from **100c.**

Yields varied between 20 and 50% and it was postulated that the forming chalcones acted as internal filters and prevented complete conversion of the cinnamates. No *cis*-chalcones were isolated. After confirming that the photo-Fries reaction proceeded with the sample ester **100a**, which yielded **101a** as the major rearrangement product, different substituents were introduced on the A ring and it was found that the 2-methoxy- (compound **100b**) and 3-hydroxy- (compound **100c**) phenylcinnamates yielded the products of *ortho* migration, namely **101b** from **100b** and a mixture of **101c** and **101d** from **100c**. Finally the set of compounds **100e-k** was photolysed to get **101e**-**101k**. The photo-Fries reaction of the phloroglucinol mono-ester is particularly simple since, for reasons of symmetry, the two products of *ortho* migration and that of *para* migration are identical.

**Scheme 42:**
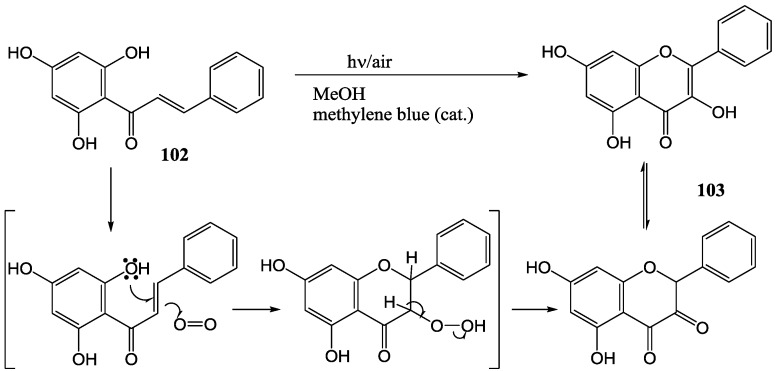
Proposed mechanism of the photo-oxygenation of chalcones.

Chawla and Chibber [77] studied the photo-oxygenation of chalcones. Irradiation of 2',4',6'-trihydroxychalcone (**102**) in methanol containing catalytic amounts of methylene blue under air with a 100 W tungsten lamp yielded the corresponding flavonol **103**. The suggested mechanism is given in Scheme 42. No yields were reported. No reaction took place in the absence of methylene blue, suggesting the involvement of singlet molecular oxygen. Replacement of methylene blue with rose bengal gave lower yields, probably due to a lower concentration of ^1^Δ_g _oxygen and the formation of ^1^E_g_ oxygen which leads to the formation of side products [78]. 

**Scheme 43:**
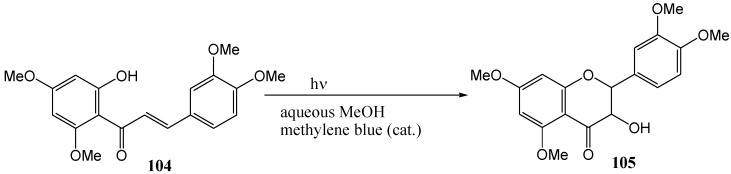
Photochemical conversion of chalcones to flavanols.

Subsequently Chawla and coworkers [79] studied the conversion of chalcones into dihydroflavonols. Irradiation of 2'-hydroxy-4',6',3,4-tetramethoxychalcone (**104**) in 4% aqueous methanol and a catalytic amount of methylene blue with a 100 W tungsten flood lamp yielded the corresponding flavanol **105** (compared to flavonol **103** in previous paper [75]) (Scheme 43), but no 2,3-relative stereochemistry was indicated. No reaction was observed in non-aqueous solvents such as benzene, benzene-methanol mixtures, or absolute methanol. Upon addition of small amounts of water (ca. 5%) the product was observed. This suggests that water is the source of the 3-OH in the flavanol product. Quinol, a well known radical quencher, inhibited the reaction, suggesting a radical mechanism. No yield was reported. The reaction was compared to the chlorophyll sensitised oxygenation in plants.

**Scheme 44:**
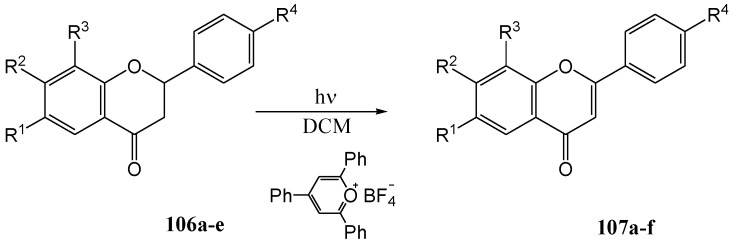
Photosensitized dehydrogenation of flavanones to flavones with 2,4,6-triphenylpyrylium tetrafluoroborate (TPT).

Climent and co-workers [80] investigated the electron-transfer dehydrogenation of flavanones. They transformed flavanones **106a-c,e** into the corresponding flavones **107a-c,e** in yields ranging from 50 to 69% (Scheme 44, Table 13) using triphenylpyrilium tetrafluoroborate (TPT) in dichloromethane with a Pyrex immersion well and 125 W medium pressure lamp (potassium chromate solution as filter). 

**Table 13:** The effect of substituents on the yield of photosensitized dehydrogenation of flavanones to flavones with 2,4,6-triphenylpyrylium tetrafluoroborate (TPT) (R^1^ – R^4^ substituents on **106** and **107**).

Substituent	Yield (%)
**a**, R^1^ = R^2^ = R^3^ = R^4^ = H	59
**b**, R^2^ = OMe, R^1^ = R^3^ = R^4^ = H	68
**c**, R^4^ = OMe, R^1^ = R^2^ = R^3^ = H	69
**d**, R^4^ = NO_2_, R^1^ = R^2^ = R^3^ = H	0
**e**, R^1^ = R^3^ = Me, R^2^ = R^4^ = H	52*

* **107f** (13%) was also obtained (R^1^ = CHO, R^2^ = R^4^ = H, R^3^ = Me).

**Scheme 45:**
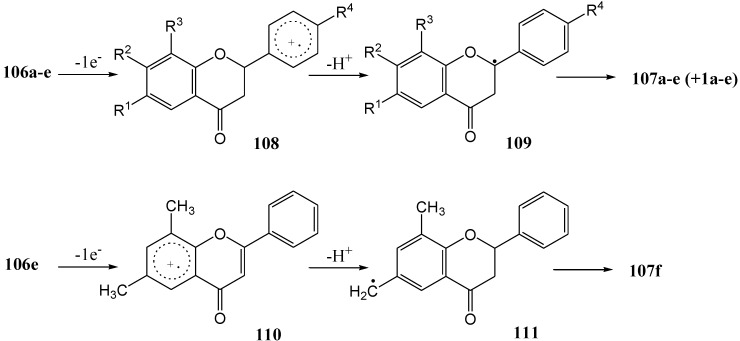
Proposed single electron transfer mechanism of photosensitized dehydrogenation of flavanones to flavones with 2,4,6-triphenylpyrylium tetrafluoroborate (TPT).

4'-Nitroflavanone **106d** was inert under the reaction conditions. The mechanism was rationalised in terms of an initial single electron transfer from the aromatic B-ring of the flavanone to the excited pyrilium salt to give the radical cation **108** or **110** which disproportionated to the flavones. Prevention of the reaction by an electron-withdrawing nitro group and acceleration by electron-donating methoxy substituents supports this mechanism (Scheme 45). 

## 4. Chalcone Epoxides

**Scheme 46:**
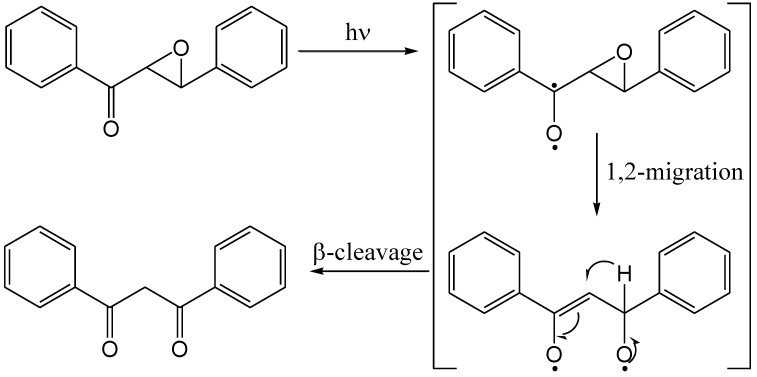
Proposed mechanism for photochemical β-diketone formation from α,β-epoxychalcone.

Bodforss [81] epimerised 4'-chloro-α,β-epoxychalcone to the corresponding β-diketone in benzene with sunlight. A mechanism was proposed by Zimmerman [82] for a non-chlorinated derivative involving C_α_-O ring-opening (Scheme 46). 

**Scheme 47:**
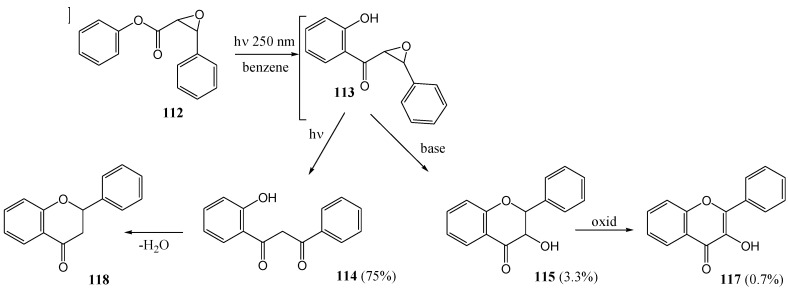
Photochemical conversion of phenyl epoxycinnamate to flavonoids.

Ramakrishnan and Kagan [83] irradiated the phenyl epoxycinnamate **112** at 250 nm (in benzene under nitrogen) and obtained the 2-hydroxybenzoylacetophenone (β-diketone) **114** in 75% yield. This β-diketone was readily converted to the corresponding flavone **118** with sodium acetate in acetic acid. The same β-diketone **114** was obtained upon irradiation of phenyl epoxycinnamate (**112**). It was postulated that 2'-hydroxyepoxychalcone **113** was an intermediate in this reaction, *via* a photo-Fries rearrangement. The isolation of small quantities of dihydroflavonol **115** (3.3%) and flavonol **117** (0.7%) was explained in terms of thermal rearrangements and oxidation of **113** (Scheme 47). No 2,3- relative stereochemistry was indicated for **115**. Phenol (16%) and stilbene (10%) were also isolated and a carbene mechanism was suggested. Efforts to broaden the scope of this reaction failed because other substituted phenyl cinnamates could not be converted to their corresponding epoxides.

**Scheme 48:**
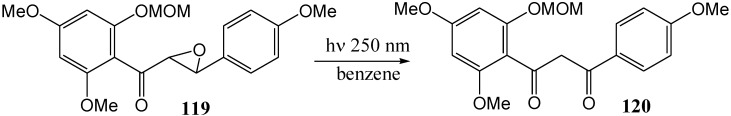
Photochemical transformation of 4,4',6'-trimethoxy-2'-methylmethoxy-*trans* chalcone-epoxide to the corresponding β-diketone.

Van der Westhuizen and co-workers [84] irradiated 4,4',6'-trimethoxy-2'-methylmethoxy-*trans* chalcone-epoxide (**119**) in benzene at 250 nm under nitrogen and obtained the anticipated [85] β-diketone **120** (32%) *via* heterocyclic C_α_-O heterolysis and hydrogen transfer (Scheme 48). 

**Scheme 49:**
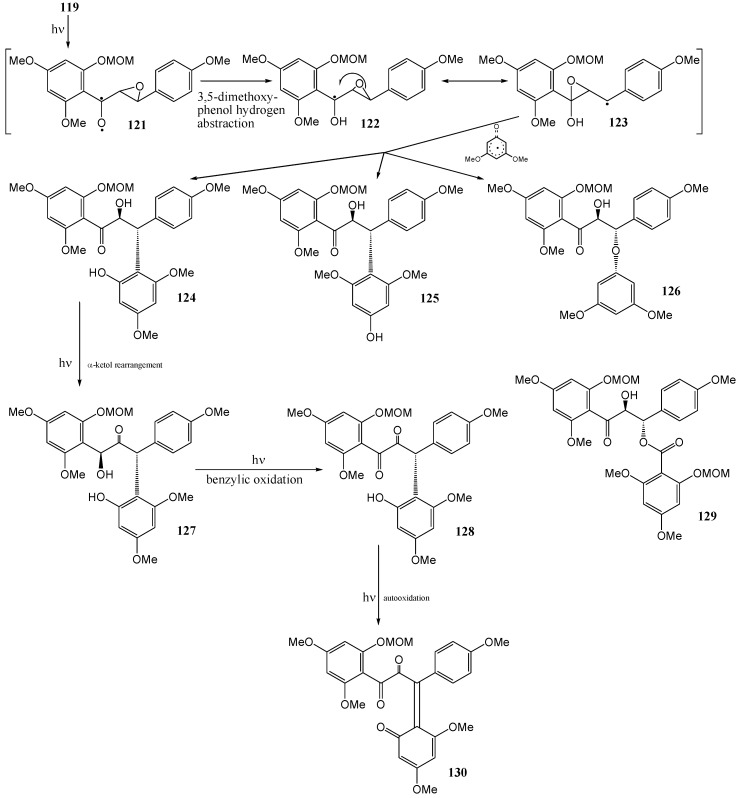
C_β_ coupled analogues obtained from irradiation of 4,4',6'-trimethoxy-2'-methylmethoxy-*trans* chalcone-epoxide.

Repetition of the reaction under identical conditions except for the presence of 3,5-dimethoxyphenol, afforded a complex mixture (47% overall yield) and the C_β_-C coupled analogues (*via* C_β_-C homolysis) **124**, **125**, **127**, **128**, and **130**, C_β_-*O* coupled isomer **126**, and a C_β_-O linked ester **129** were isolated (Scheme 49). 

Formation of the β-coupled products **124 -126** was explained in terms of hydrogen abstraction from the phenol by an n,π*-excited carbonyl, rearrangement of the radical **122** to the corresponding benzyl radical **123**, and coupling with the associated phenoxy radical (probably in a cage) at the 2-, 4-, or oxygen position. The β-coupled benzoyl ester **129** has a similar origin and indicates photolytic formation of a benzoic acid analogue. Photochemical α-ketol rearrangement *via* photoenolisation of **124** explained the formation of the propan-2-one **127**. Oxidation of **127** yielded structures **128** and **130**.

## 5. Reactions Initiated *via* Hydrogen Abstraction by an Excited State Carbonyl

The excited state carbonyl group, generally assumed to be ^3^(n,π*), may abstract a suitably positioned hydrogen to form a biradical intermediate that may undergo further rearrangements to novel products. Photochemical keto-enol isomerisation and further transformations of the enol is included in hydrogen abstraction by carbonyls. 

**Scheme 50:**
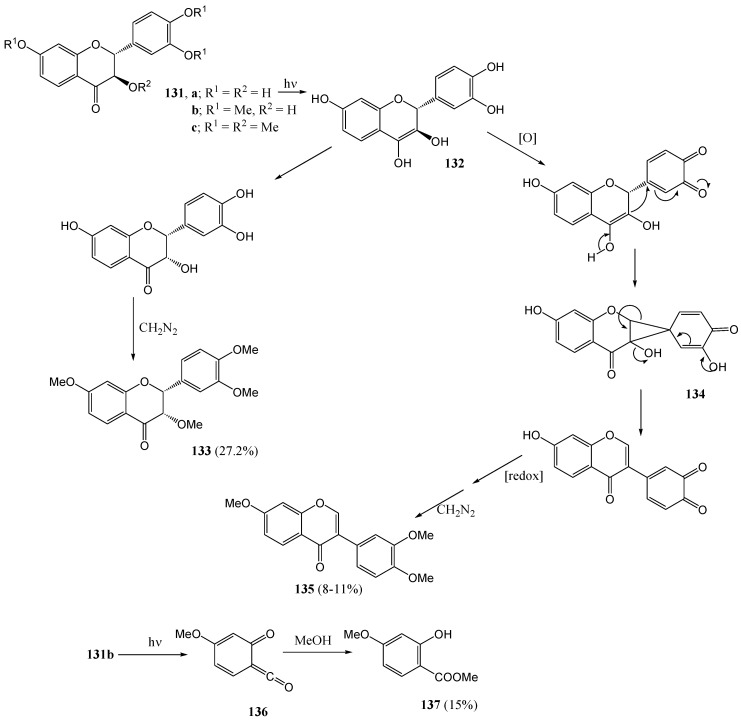
Photochemistry of 2,3-*trans*-flavanols.

Fourie and co-workers [86] studied the photochemistry of 2,3-*trans*-flavanols **131a-c**. Irradiation of free-phenolic (+/-)-2,3-*trans*-fustin (**131a**) at 300 nm in methanol yielded the thermodynamically less stable 2,3-*cis* isomer **133** (27%) after complete methylation, and the isoflavone analogue **135** after final reduction and methylation. Both these products may form *via* an enol intermediate **132**. The tri-*O*-methylether **131b** gave none of the products obtained from free-phenolic fustin **131a**, but instead underwent homolysis of both the heterocyclic O-C and α-carbonyl bonds (Norrish type I process) to yield methyl-2-hydroxy-4-methoxybenzoate **137***via* trapping of the intermediary ketene **136** (25%) (Scheme 50). In contrast to the products obtained from free-phenolic and tri-*O*-methyl fustin, the tetra-*O*-methyl analogue **131c** gave 2'-hydroxy-3,4,4'-trimethoxy-*trans*-chalcone (**138**, 58%) and tri-*O*-methylflavanone **139** (34%), as shown in Scheme 51. The mechanism requires abstraction of a hydrogen atom from the 3-methoxy group by the excited carbonyl chromophore, followed by formaldehyde loss. The absence of the *cis*-chalcone suggested slow *trans*-*cis* isomerisation, probably due to inefficient light absorption at 300 nm.

**Scheme 51:**
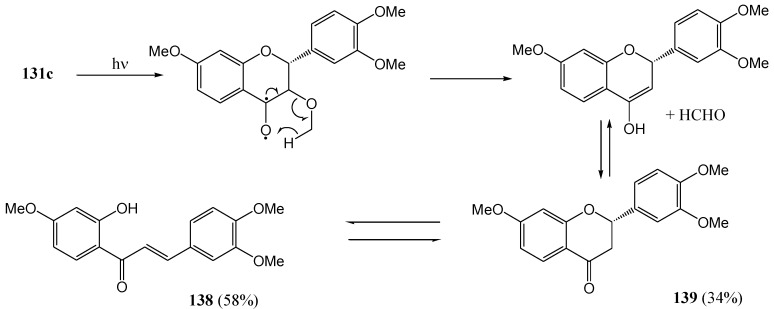
Photochemistry of tetra-*O*-methyl fustin (permethylated flavanol).

Van der Westhuizen and co-workers [87] described the photochemical deoxygenation of the series of flavanones depicted in Figure 5. 

**Figure 5:**
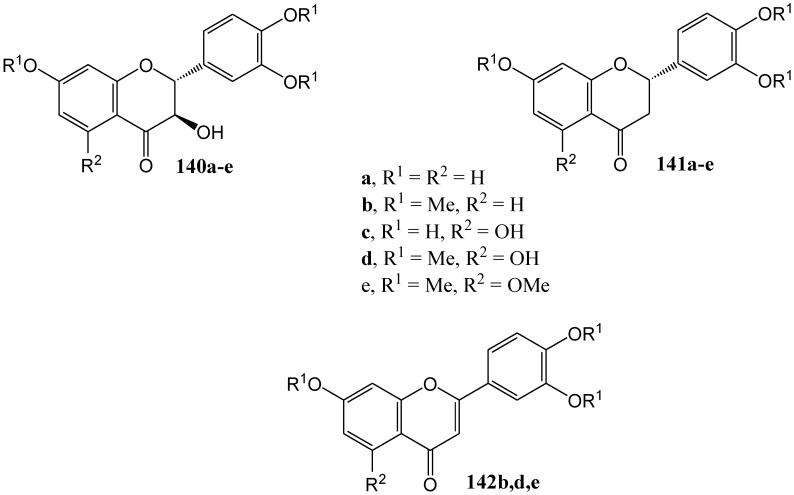
Flavanones and flavone obtained from flavanols.

Irradiation of (+)-fustin (**140a**) at 350 nm in dry ethyl acetate under nitrogen yielded the flavanone (-)-butin (**141a**) (2*S*) (37%). In the case of tri-*O*-methylfustin (**140b**) both the corresponding flavanone **141b** (21%) and flavone **142b** (15%) were isolated. (-)-Fustin gave the flavanone with the inverse configuration (2*R*). Addition of phloroglucinol as a hydrogen donor increased the yield of **141a** to 47%, of **141b** to 45%, and of **142b** to 28%. Naphthalene, as triplet quencher, reduced the yield of **141a** to 7% and increased the yield of the flavone **142b** to 53%. 

**Scheme 52:**
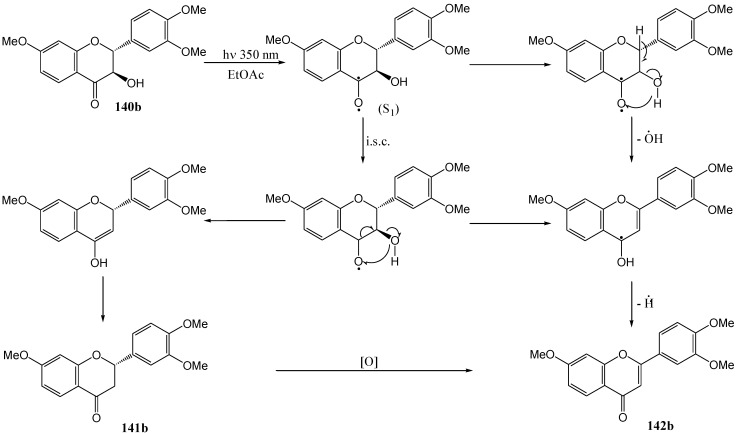
Photochemical deoxygenation of an α-ketol to a flavone.

These results indicate that deoxygenation takes place from an n,π*-excited state *via* hydrogen abstraction. Phloroglucinol probably enhances this process. Efforts to transform the flavanone **141b** into the flavone photolytically led to only minor transformation to the flavone **142b** (6%). It would appear that the flavanone **141b** is not an intermediate in the formation of **142b** from **140b**. Nascent oxygen liberated during deoxygenation may be responsible for the oxidation of **141b** to **142b** (Scheme 52). The low conversion of the flavanone **141b** to the isomeric *trans*-chalcone **143** in ethyl acetate (Scheme 53) contrasts with the results in polar solvents (MeOH) where the equilibrium is towards the chalcone (Scheme 51) [86]. This allows isolation of optically pure flavanone **141b**.

**Scheme 53:**
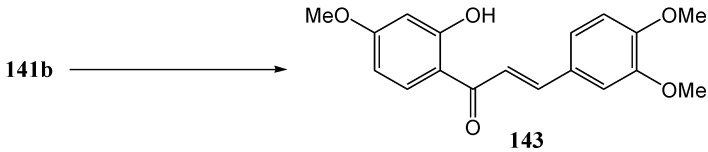
Photochemical conversion of flavanone to *trans*-chalcone.

(+)-Tri-*O*-methyldihydroquercetin (**140d**) has a hydroxyl group at C-5 that may form a hydrogen bond with the carbonyl chromophore thus resisting photo-transformation, and hampering the general applicability of the method. Efforts to overcome the problem with triplet sensitizers gave poor results (2%), but addition of phloroglucinol increased the yields of the deoxygenated product **141d** to 23%.

Van der Weshuizen and co-workers [88] investigated the photochemical methoxy-hydroxymethyl isomerization of 4-methoxybenzo[b]furan-3(2*H*)-ones **144a** and **144b**. Irradiation of 2-benzyl-2-hydroxybenzo[*b*]furan-3(2*H*)-one (**144a**) at 350 nm in anhydrous ethyl acetate under nitrogen yielded **149** (21%) and **150** (16%). Irradiation of **144b** yielded **147b**. These products require methoxy-hydroxymethyl isomerisation of the 4-methoxy substituent (Scheme 54). 

**Scheme 54:**
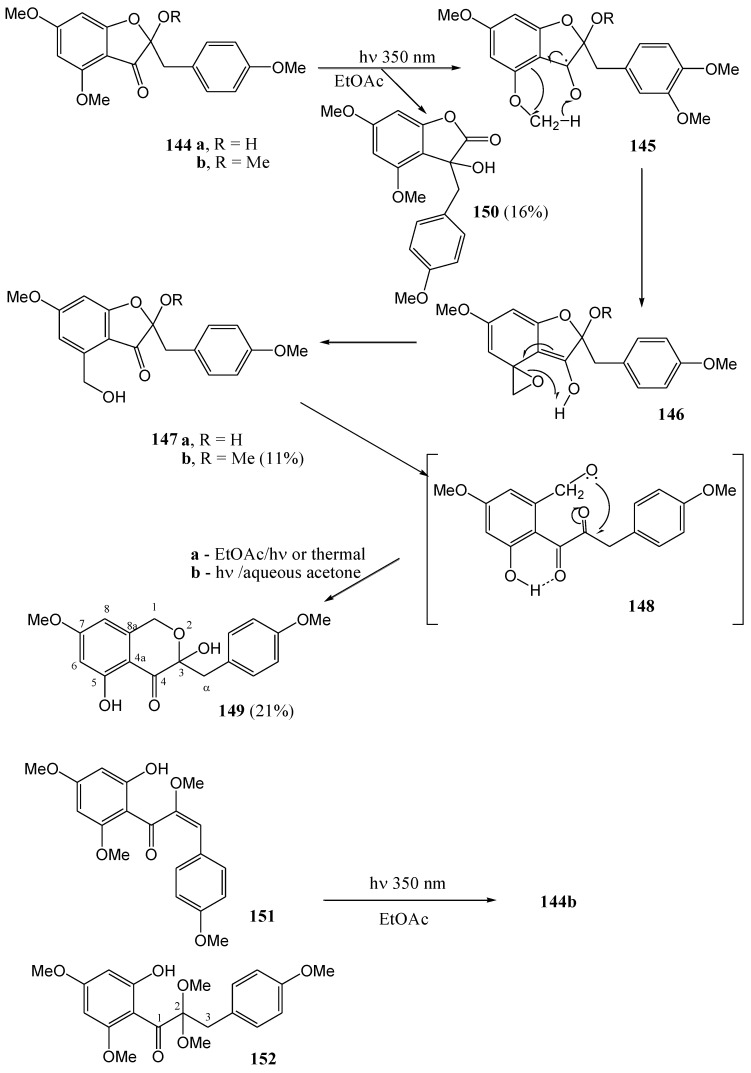
Photochemical methoxy-hydroxymethyl isomerization of 4-methoxy-benzo[b]furan-3(2*H*)-ones.

In **144a** the 2-hydroxy allows incorporation of the aromatic -CH_2_OH group in the heterocyclic ring that changes the five-membered to a six-membered ring. In the case of the fully methylated 2-methoxybenzo[*b*]furan-3(2*H*)-one (**144b**), the five-membered ring resisted ring-opening and the product with a free -CH_2_OH (compound **147b**) was isolated (11%). It was postulated that the benzylic acid rearrangement has ionic character and takes place from a π,π* excited state which has ionic character and is encouraged by polar solvents [89]. Formation of **150** is described in Scheme 60 [90].

## 6. Reactions of Flavonoids with a Fully Saturated C-Ring (no Carbonyl Chromophore). Benzyl Ether Fission

Benzyl ethers typically undergo photolytic fission of the C-O bond. This is assisted by the stability of the benzylic radical or ionic intermediates. The heterocyclic C-ring of flavonoids contains an intramolecular benzylic ether bond. 

**Scheme 55:**
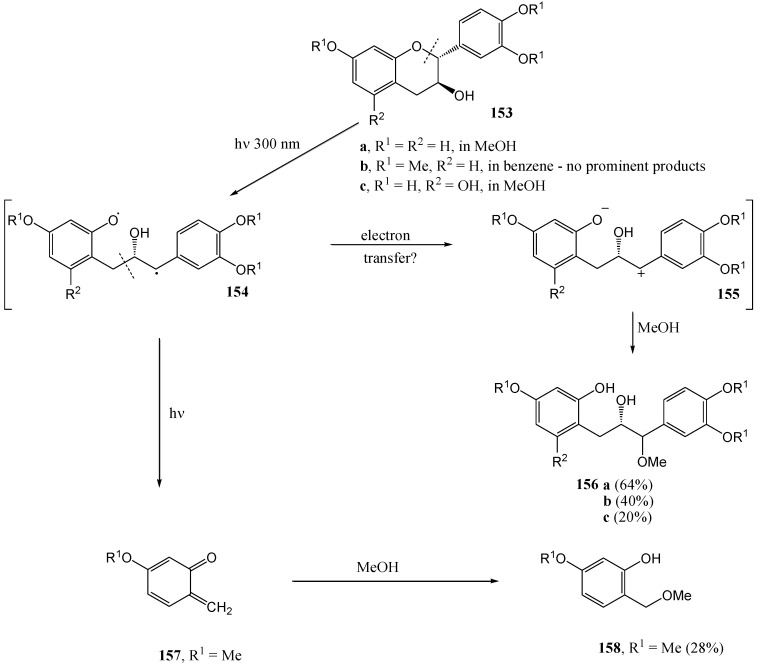
Photochemistry of flavan-3-ols.

Fourie and co-workers [86] studied the photochemistry of flavan-3-ols. Irradiation of free phenolic flavan-3-ol **153c**, fisetinidol (**153a**) and its tetra-*O*-methyl ether **153b** at 300 nm in methanol yielded the corresponding optically active 1,3-diarylpropan-2-ols **156a** and **156b** (in 64 and 40% yield, respectively). The mechanism involves fission of the heterocyclic benzylic *O*-C bond and trapping of the resulting intermediary benzylic carbocation **155** with methanol.

The methyl ether **153b** also yielded 5-methoxy-2-methoxymethylphenol (**158**), *via* subsequent fission of the C3-C4 bond of radical **154** and trapping of the resulting *ortho* quinone methide **157** with methanol. The absence of the free phenolic analogue of **157** probably indicates stabilization of the free-phenolic benzylic carbocation as a *para*-quinone methide. Irradiation of the tetra-*O*-methyl ether **153b** in benzene did not give any product. Benzene is probably not sufficiently polar to stabilise the intermediate carbocation **155** (Scheme 55). Under identical conditions free-phenolic catechin **153c** gave the corresponding diarylpropan-2-ol **156c** (20%). Irradiation of flavan-3-ols results in homolysis of the heterocyclic 1,2-(O-C) and 3,4-(C-C) bonds.

Van der Westhuizen and co-workers [91] studied the photochemistry of tannin analogues. Benzophenone sensitized photolysis of free phenolic (2*R*,3*S*,4*S*)-2,3-*trans*-3,4-*trans*-4-(2,4,6-tri-hydroxyphenyl)-flavan-3-ol (**159**) at 350 nm in acetone yielded (2*S*,3*S*,4*S*)-2,3-*cis*-3,4-*cis*-4-(2,4-dihydroxyphenyl)-flavan-3-ol (**160**). 

**Scheme 56:**
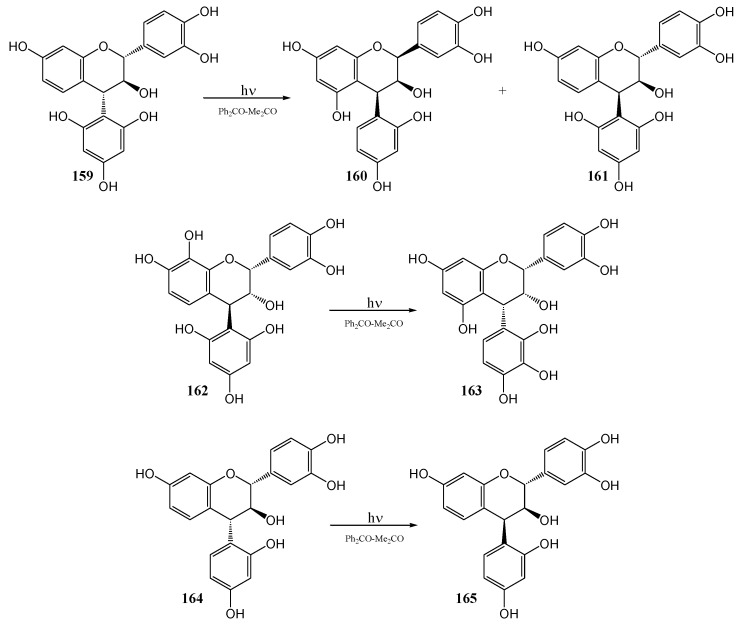
Synthesis of 2,3-*cis*-3,4-*cis* 4-arylflavan-3-ols by photolytic rearrangement.

The mechanism involves formal cleavage of the heterocyclic ether bond followed by recyclisation *via* the quinone methide-stabilized benzylic carbocation **166** involving attack by the more nucleophilic phloroglucinol D-ring (isomerisation of the A-and D-ring) on the α-face of C-2 (*anti* to the 3-hydroxy substituent). 

Inversion of configuration at C4 is thus a result of rotation about the C3-C4 bond and inversion of configuration at C2 is probably controlled by the orientation of the 3-OH substituent *via* a protonated oxirane intermediate **169**. Traces of the (2*R*,3*S*,4*R*) 2,3-*trans*-3,4-*cis*-4-(2,4,6-trihydroxyphenyl)-flavan-3-ol (**163**), were also isolated. 

**Scheme 57:**
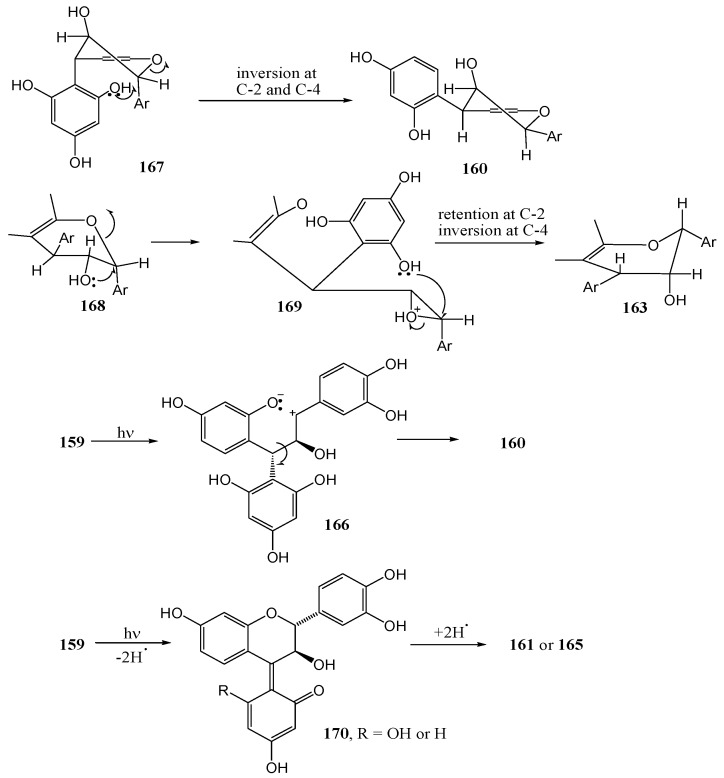
Proposed mechanism for photochemical inversion of configuration at C-4 of 4-arylflavan-3-ols.

Formation of **161** involves no ring-isomerisation but only inversion of configuration at C4, probably *via* an A-ring quinone methide yielded the (2*R*,3*R*,4*R*) 2,3-*cis*-3,4-*cis* product **163**. The stereochemistry at C4 was determined by rotation about the C3-C4 bond and configuration at C2 by the orientation of the hydroxy group on C3. With a weak nucleophilic D-ring, isomerisation of the A- and D-ring is not observed. Photolysis of (2*R*,3*S*,4*R*) 2,3-*trans*-3,4-*trans*-4-(2,4,-dihydroxyphenyl)-flavan-3-ol (**164**) yielded the (2*R*,3*S*,4*S*) 2,3-*trans*-3,4-*cis* isomer **165**. Inversion of configuration at C4 probably takes place *via* an A-ring quinone methide, similar to **170** described above. The reactions are given in Scheme 56 and a suggested mechanism provided in Scheme 57.

## 7. Photochemistry of 2-Benzylbenzofuranones

**Scheme 58:**
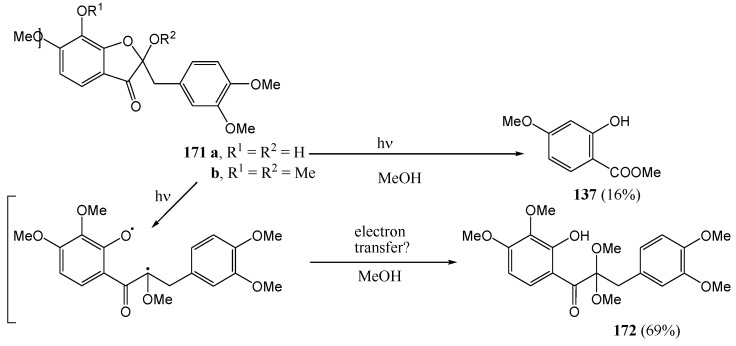
Photochemistry of 2-benzylbenzofuranones.

Fourie and co-workers [86] studied the photochemistry of 2-benzylbenzofuranones in methanol at 300 nm in quartz vessels. Photofragmentation of the heterocyclic *O*-C and α-carbonyl bonds of the tri-*O*-methyl-2-benzyl-2-methoxybenzo[*b*]furan-3(2*H*)-one (**171a**) yielded 2-hydroxy-4-methoxybenzoate (**137**, 16%), similar to tri-*O*-methylfustin (see also Scheme 51). In contrast, bond fission in the tetra-*O*-methyl-2-benzyl-2-methoxybenzo[*b*]furan-3(2*H*)-one (**171b**) equivalent is restricted to the *O*-C bond and the 2,2-dimethoxyacetal **172** (69%) is isolated *via* solvolysis of the ring-opened intermediate (Scheme 58). 

**Scheme 59:**
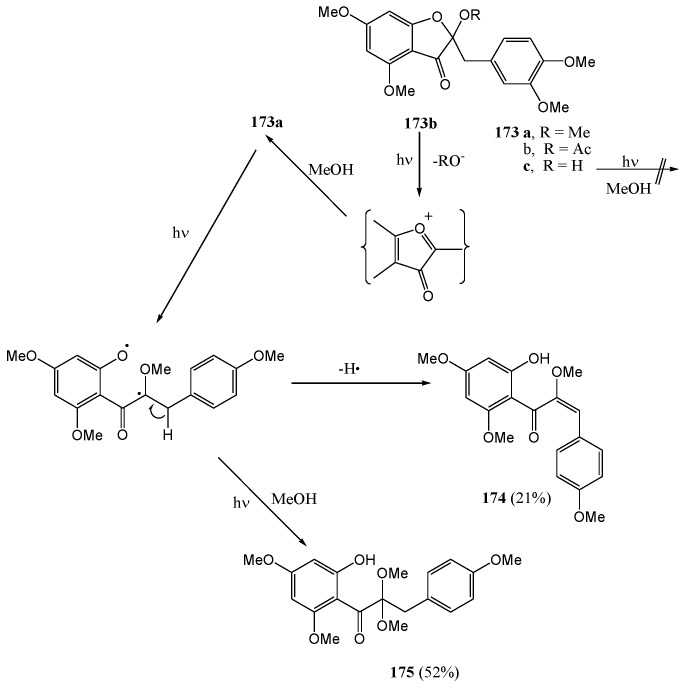
Photochemistry of 2-benzylbenzofuranones.

The 2-benzylfuranone (**173a**) where the B-ring has 4-methoxy- rather than 3,4-dimethoxy-substitution and the A-ring is of the phloroglucinol-type, yielded a *cis*-α-methoxychalcone (**174**) (21%) besides the corresponding acetal **175** (52%). The 2-acetoxy derivative **173b** gave the same acetal as the α-methoxychalcone while the 2-hydroxy derivative **173c** did not react (Scheme 59). By contrast, photofragmentation of 2-benzyloxyfuranones under the same conditions is confined to the heterocyclic C-O bond to form acetals **172** and **175**.

Van der Weshuizen and co-workers (1977) [90] found that irradiation of 2-benzyl-2-methoxy-benzo[*b*]furan-3(2*H*)-one (**144b**) at 350 nm in acetone–water, dioxane-water or tetrahydrofuran-water resulted in isolation of the 2-hydroxy derivative **144a** (42%) and small amounts of the corresponding α-methoxy-*cis*-chalcone **151** (15%). In the absence of water the α-methoxy-*cis*-chalcone **151** (60%) was the only product. 

**Scheme 60:**
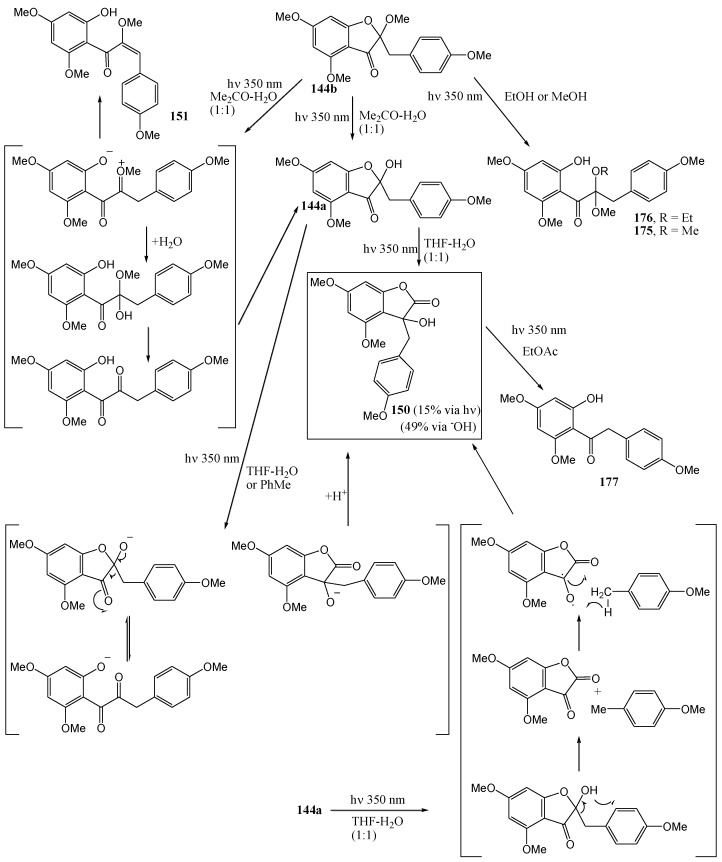
Photochemical equivalent of benzilic acid rearrangement and related conversions.

Irradiation of the 2-hydroxy derivative **144a** at 350 nm resulted in a unique photochemical equivalent of the benzilic acid rearrangement to give the 3-benzyl-3-hydroxybenzo[*b*]furan-2(3*H*)-one analogue (**150**) (15%). The proposed mechanism involves an α-diketone and an 1,2-shift. Irradiation of (**150**) [88] in anhydrous ethyl acetate results in decarbonylation of the heterocycle *via* a Norrish type I process, leading to the deoxybenzoin **177** (Scheme 60).

## 8. 4-Phenylchroman-3-one

**Scheme 61:**
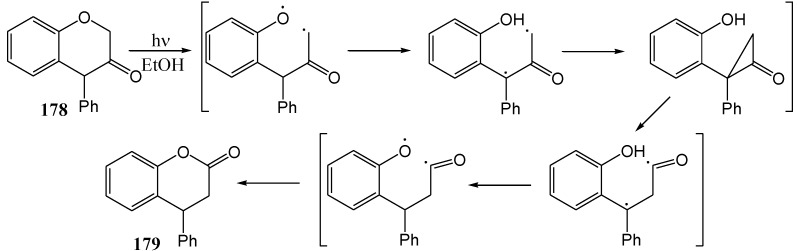
Proposed mechanism for the photochemical rearrangement of 4-phenyl-3-chromanone to 4-phenyldihydrocoumarin.

The photochemistry of these compounds is of interest because of the C4 diaryl functionality that appears in all proanthocyanidins. Grover and Anand [92] transformed 4-phenyl-3-chromanone (**178**) to 4-phenyldihydrocoumarin (**179**, 35%) in ethanol using a Hanovia 450 W mercury lamp (Scheme 61). The proposed mechanism occurs *via* intramolecular hydrogen abstraction *via* a five-membered cyclic transition state.

**Scheme 62:**
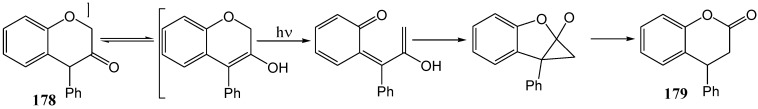
Alternative mechanism for the photochemical rearrangement of 4-phenyl-3-chromonone to 4-phenyldihydrocoumarin (enol tautomer intermediate).

**Scheme 63:**
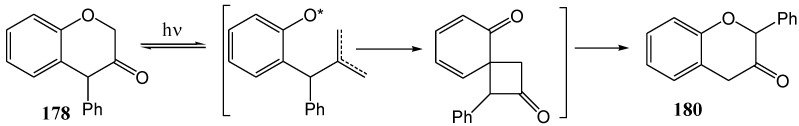
Proposed mechanism for the photochemistry of 4-phenylchroman-3-one in acetonitrile or benzene.

Later Padwa and Lee [93] suggested an alternative mechanism involving an enol tautomer (Scheme 62). Padwa and Au [94], and also Padwa and co-workers [95], subsequently repeated the reaction in benzene or acetonitrile to obtain 2-phenylchroman-3-one (**180**) in 60% yield and the proposed mechanism explaining this different product is outlined in Scheme 63. They demonstrated tautomer control *via* use of the appropriate solvent. 

## 9. Diverse Reactions

**Scheme 64:**
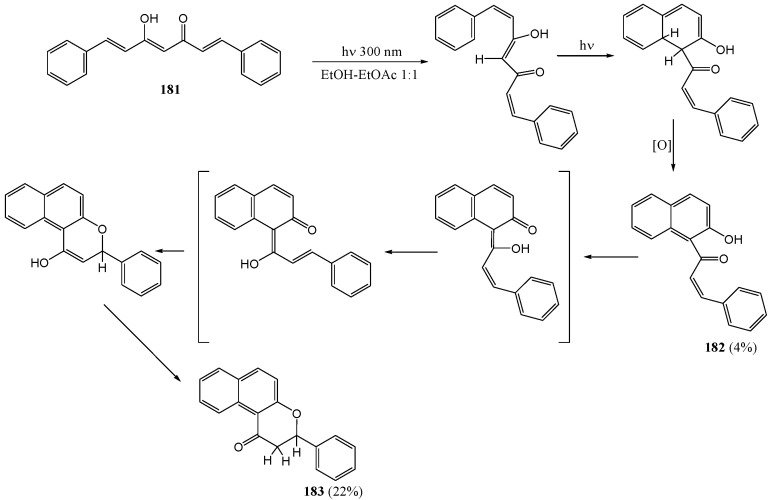
Photochemistry of 1,7-diphenyl-1,6-heptadiene-3,5-dione.

Sundaryoano and co-workers [29] investigated the photochemistry of 1,7-diphenyl-1,6-heptadiene-3,5-dione, a non-phenolic curcuminoid model. Upon irradiation of unsubstituted curcumin (**181**) with a medium-pressure mercury lamp (400 W) in an ethanol-ethyl acetate mixture they obtained 2'-hydroxy-5',6'-benzochalcone **182** (4%), the corresponding flavanone **183** (22%), benzaldehyde, and cinnamaldehyde (Scheme 64). 

Formation of the chalcone **182** was postulated to take place *via* photocyclisation of the excited triplet state of the enol form of **181** followed by a thermal oxidative step. Formation of the flavanone **183** was assumed to be *via* photochemical cyclisation in agreement with the work by Matshushima and coworkers (1985) [67]. This work represents a unique example of the photochemical conversion of a molecule from one important class of natural products (diarylheptanoids) into another important class (flavonoids).

**Scheme 65:**
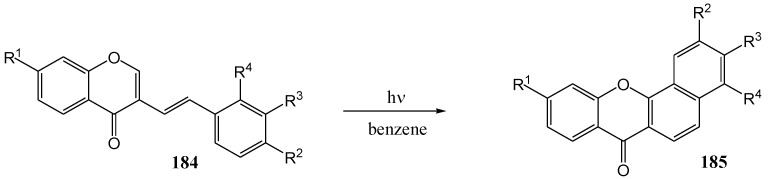
Photocyclization of 2-styryl-4*H*-chromen-4-ones.

Yokoe and co-workers [96] studied the photochemistry of 2-steryl-4*H*-chromen-4-ones **184**. Irradiation of **184** in benzene with a high-pressure mercury lamp at room temperature under air yielded benzo[*a*]xanthones **185** (Scheme 65) *via* cyclization in yields of between 43 and 88%, depending on the aromatic substituents (Table 14). In the case of **184d** and **184e**, there are two possible directions of cyclization, *ortho* or *para* to the methoxy group on the benzene ring. However, the photocyclized products (**185d** and **185e**) each showed a single spot on TLC. This reaction provides a general route to polycyclic xanthene derivatives.

**Table 14:** Effect of substituents on the yield of the photocyclization of 2-styryl-4*H*-chromen-4-ones.

184	R^1^	R^2^	R^3^	R^4^	Yield (%) of 185a-i
a	H	H	H	H	22
b	H	Me	H	H	23
c	H	OMe	H	H	1
d	H	H	OMe	H	15
e	H	OMe	OMe	H	7
f	Me	H	H	H	11
g	Me	Me	H	H	13
h	Me	OMe	H	H	2
i	H	H	H	Br	31

**Scheme 66:**
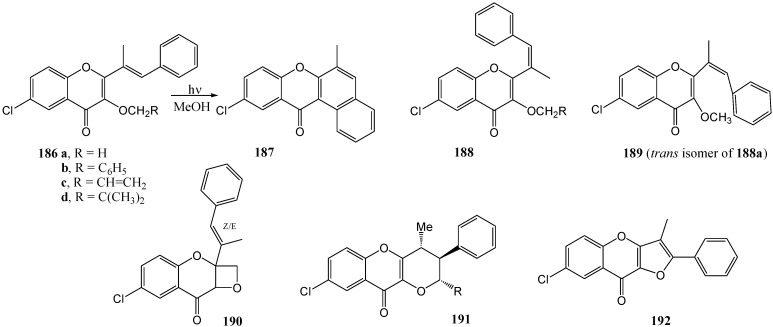
Photochemistry of some 3-alkoxy-2-sterylchromones.

Kamboj and co-workers [97] observed similar results upon irradiation of 3-alkoxy-2-styryl-chromones **186a-d** with a 125 W mercury vapour lamp in methanol under nitrogen to afford six different structures **187**-**192** (Scheme 66, Table 15). 

**Table 15:** Effect of substituents on the photochemistry of 3-alkoxy-2-sterylchromones.

	Yield (%)				
186	187	188	190	191	192
**a**, R = H	6	54	5	8	
**b**, R = C_6_H_5_	7	55		10	3.0
**c**, R = CH=CH_2_	8.5	56		14	10
**d**, R = CH=C(CH_3_)_2_	9	58		7	19

**Scheme 67:**
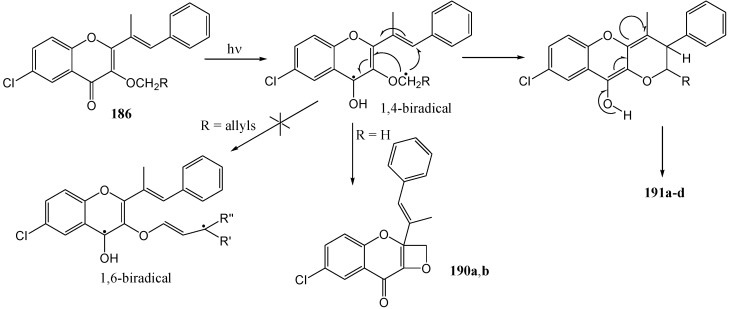
Suggested mechanism for the formation of the photoproduct from 3-alkoxy-2-sterylchromones.

The suggested mechanism for the formation of the photoproduct through dealkylation and excited state intramolecular proton transfer is given in Scheme 67. 

**Scheme 68:**
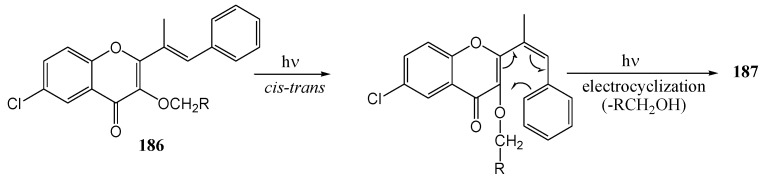
Suggested mechanism for the cyclisation of 3-alkoxy-2-styrylchromones.

**Scheme 69:**
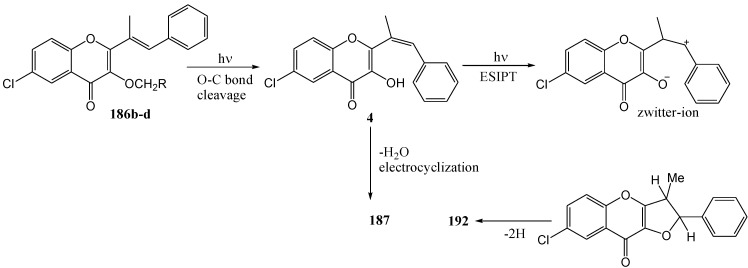
Suggested mechanism for the dealkylation and excited state intramolecular proton transfer of 3-alkoxy-2-styrylchromones.

Scheme 68 shows the mechanism *via* isomerisation and cyclisation and Scheme 69 represents formation of the photoproduct through dealkylation and excited state intramolecular proton transfer.

**Scheme 70:**
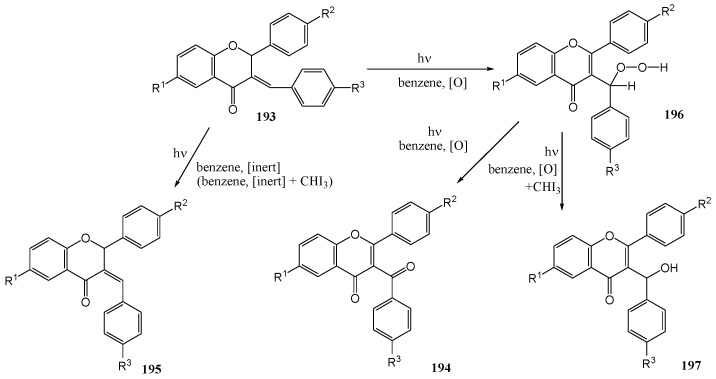
Phototransformation of 3-arylideneflavanones to 3α-hydroxybenzylflavones.

Dhande and co-workers [98] investigated the photochemistry of *E*-3-benzylideneflavanones **193**. Aroylflavone **194** were obtained upon irradiation of compound **193** in dry benzene in the presence of air or oxygen (125 W high-pressure mercury lamp in quartz) as given in Scheme 70. Yields varied between 27 and 90% depending on the substituent on the aromatic rings. Isomerisation of the double bond to *Z*-3-benzylideneflavanones **195** was observed in all cases. Under inert atmosphere only the isomerisation product **195** was observed. As the *Z*-isomer with the C-2 aryl in the equatorial position has the H-2 proton in a suitable axial position for a concerted ene reaction with singlet oxygen, it was suggested that isomerisation to the Z-isomer preceded oxidation to a hydroperoxide intermediate **196**. Efforts to increase the reaction rate with rose bengal (a singlet oxygen source), however, failed. In dichloromethane with a phase transfer catalyst and rose bengal or methylene blue, no oxidation and only *E* to *Z* isomerisation was observed. Yields for each different derivative are given in Table 16. 

Halogen containing solvents (such as chloro-, bromo-, and iodobenzene) or addition of iodoform reduced the isomerisation time under nitrogen. Addition of iodoform to the benzene reaction mixture under air gave 3-α-hydroxybenzylflavones **197** upon photolysis using a Pyrex immersion well in yields of between 60 and 88% depending on the substituent. This represents a general route to these otherwise unavailable compounds. Arylideneflavanones **193** upon UV irradiation using quartz undergo auto-oxidation to 3-aroyl-flavones **194**. Photolysis by using a Pyrex filter in the presence of iodoform furnishes 3-α-hydroxy-benzylflavones indicating the intermediacy of the hydroperoxide **196** and represents a new general method for the synthesis of these compounds not available by other routes. Under the inert conditions the irradiation furnishes the isomerised product **195**.

**Table 16:** Effect of substituents on the yields of phototransformation of 3-arylideneflavanones to 3α-hydroxybenzylflavones.

193,194,197	a	b	c	d	e	f	g	h	i
R^1^	Me	Me	Me	H	H	H	H	H	H
R^2^	OMe	Cl	OMe	H	H	H	H	H	H
R^3^	Cl	H	OMe	H	Me	OMe	OCH_2_Ph	Cl	NO_2_
**194**, Yield (%)	32	27	31	90		81	86	81	
**197**, Yield (%)				65	66	61	60	70	88

**Scheme 71:**
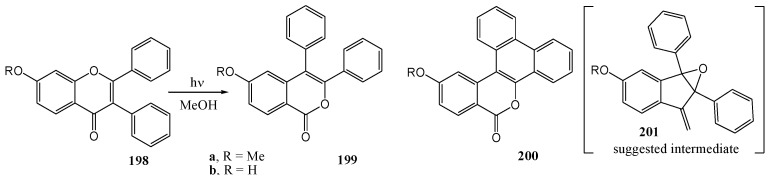
Photochemistry of 2-phenylisoflavones.

Ishibe and co-workers (1975) [99] irradiated 2-phenyl-7-methoxyisoflavone (**198a**) in methanol with a medium-pressure mercury lamp and a Pyrex filter and obtained the corresponding 3,4-diphenyl-isocoumarin **199a** (10%) and a pentacyclic structure **200a** (42%), presumably *via* intermediate **201**. 2-Phenyl-7-hydroxyisoflavone was unreactive in air but gave (**200b**) in the presence of iodine. Photo-isomerisation was not observed with 2-methyl-7-hydroxyisoflavone and 2-methylisoflavone in methanol, indicating that the presence of a 2-phenyl-substituent was a prerequisite (Scheme 71).

## 10. Conclusions

A plethora of photochemical transformations of flavonoids has been described over the past 50 years [100] and much progress has been made to better understand the reaction mechanisms and the associated influence of reaction conditions on yields and products. Yet photochemistry still presents the researcher with many challenges and opportunities. Ephemeral goals like chemical trapping of sunlight energy to replace fossil fuels (derived from sunlight) and industrial feedstocks (also derived from sunlight) will receive considerable attention. More humble goals such as the publication of novel flavonoid photochemical transformations will keep academics active and expand our knowledge of flavonoid chemistry [101]. Photochemistry in chiral environments [102], including the use of chiral light, promises enantiomerically pure products. The use of monochromatic laser light will allow selective excitation of target chromophores. The use of high energy lasers in combination with a flow reactor will reduce the time that reagents are exposed to UV light to minimize unwanted side reactions and increase yields. Low temperature photochemistry should also yield interesting results.
